# Comparative Genomics of *Microbacterium* Species to Reveal Diversity, Potential for Secondary Metabolites and Heavy Metal Resistance

**DOI:** 10.3389/fmicb.2020.01869

**Published:** 2020-08-04

**Authors:** Erika Corretto, Livio Antonielli, Angela Sessitsch, Christoph Höfer, Markus Puschenreiter, Siegrid Widhalm, Karivaradharajan Swarnalakshmi, Günter Brader

**Affiliations:** ^1^Bioresouces Unit, Center for Health & Bioresources, AIT Austrian Institute of Technology GmbH, Tulln, Austria; ^2^Institute of Soil Research, Department of Forest- and Soil Sciences, University of Natural Resources and Life Sciences, Vienna, Austria; ^3^Division of Microbiology, Indian Agricultural Research Institute, New Delhi, India

**Keywords:** heavy metals, plant associated bacteria, comparative genomics, polyketide synthases, siderophore, terpenoids, secondary metabolites

## Abstract

*Microbacterium* species have been isolated from a wide range of hosts and environments, including heavy metal-contaminated sites. Here, we present a comprehensive analysis on the phylogenetic distribution and the genetic potential of 70 *Microbacterium* belonging to 20 different species isolated from heavy metal-contaminated and non-contaminated sites with particular attention to secondary metabolites gene clusters. The analyzed *Microbacterium* species are divided in three main functional clades. They share a small core genome (331 gene families covering basic functions) pointing to high genetic diversity. The most common secondary metabolite gene clusters encode pathways for the production of terpenoids, type III polyketide synthases and non-ribosomal peptide synthetases, potentially responsible of the synthesis of siderophore-like compounds. *In vitro* tests showed that many *Microbacterium* strains produce siderophores, ACC deaminase, auxins (IAA) and are able to solubilize phosphate. *Microbacterium* isolates from heavy metal contaminated sites are on average more resistant to heavy metals and harbor more genes related to metal homeostasis (e.g., metalloregulators). On the other hand, the ability to increase the metal mobility in a contaminated soil through the secretion of specific molecules seems to be widespread among all. Despite the widespread capacity of strains to mobilize several metals, plants inoculated with selected *Microbacterium* isolates showed only slightly increased iron concentrations, whereas concentrations of zinc, cadmium and lead were decreased.

## Introduction

The genus *Microbacterium* belongs to the *Microbacteriaceae* family, a high GC actinobacterial taxon, and accounts for more than 90 recognized species that were isolated from a wide range of habitats and hosts (Behrendt et al., 2001; Park et al., 2008; Sharma et al., 2013; Wang et al., 2014; Hadjadj et al., 2016). Several studies associated these bacteria to metal contaminated sites. Several *Microbacterium* strains can survive in heavy metal contaminated environments (Brown et al., 2012; Fidalgo et al., 2016), reduce specific metals such as hexavalent chromium (Henson et al., 2015; Fierros-Romero et al., 2016; Kumar and Saini, 2019) and change the mobility of heavy metals in contaminated soils (Kuffner et al., 2010; Soni et al., 2013). Because of these interesting traits, they have already been used in phytoextraction trials for soil decontamination (Visioli et al., 2015).

Phytoextraction is meant to be an environment friendly remediation technique, which consists in the use of metal accumulating plants to remove metals from contaminated soils by concentrating them in the harvestable parts of the plant (Garbisu and Alkorta, 2001). The main limiting factors of this technique are the production of plant biomass and the plant metal uptake, which is influenced by the bioavailability of the metal in the soil (Wenzel, 2009). Therefore, bacteria, which can either promote plant growth/health, enhance the stress tolerance and mobilize metals, are considered as an interesting resource for the improvement of such clean-up techniques (Rajkumar et al., 2012; Sessitsch et al., 2013; Ma et al., 2016).

It is well known that members of the phylum Actinobacteria produce several secondary metabolites having diverse biological activities (e.g., siderophores, antibiotics, and pigments). Many are synthesized by polyketide synthases (PKS), non-ribosomal protein synthetases (NRPS) or hybrids of these (PKS/NRPS). PKS synthesize complex polyketide compounds and are divided in three categories based on their domain structures and synthesis mechanism (Shen, 2003). Non-ribosomal protein synthetases (NRPS) are large enzymes, which synthesize secondary metabolites of peptide origin using a wide range of building blocks. They are usually organized in modules, each containing a different catalytic domain (Marahiel, 2016; Winn et al., 2016). However, some non-modular enzymes have been characterized in biosynthetic pathways for the production of NRPS-siderophores such as enterobactin and vibriobactin (Crosa and Walsh, 2002). Siderophores and organic acids are the main compounds influencing metal availability in soils (Nett et al., 2009; Qin et al., 2010; Rajkumar et al., 2010). Beside the NRPS synthesized siderophores, several bacteria produce hydroxamate and α-hydroxyacid containing siderophores through NRPS-independent pathways as in the case of desferrioxamine (Challis, 2005).

In the last years, several reports linked *Microbacterium* species to heavy metal contaminated sites and described their potential as plant growth promoting bacteria (Lumactud et al., 2017; Sun et al., 2019). The increased interest in this taxon and the advance in genome sequencing methods resulted in a high number of *Microbacterium* genomes available in the NCBI database. For instance, the studies of Henson and Learman analyzed the genetic potential of Cr(VI) reducing *Microbacterium* spp. (Henson et al., 2015; Learman et al., 2019). However, to the best of our knowledge, a comprehensive investigation of the genus *Microbacterium* has not been published yet. In this study, we aimed to (i) perform a phylogenetic analysis and cluster the selected genomes in operational functional groups; (ii) explore the predicted secondary metabolite gene clusters; (iii) investigate their plant growth promoting traits, their ability to grow in the presence of heavy metals (zinc, lead, and cadmium) and to influence metal availability in contaminated soils.

## Materials and Methods

### Genomes, Phylogenetic Analysis and Tree Construction

All genomes classified as *Microbacterium* were downloaded from GenBank (on the 8th of May, 2020; total samples *n* = 366) and initially filtered according to a maximum number of contigs < 100 (*n* = 272), completeness (>90%) and contamination (<3%) analysis with CheckM (*n* = 240) (Parks et al., 2014). Of these, 70 *Microbacterium* genomes covering the branches of the 16S rRNA tree (Supplementary Figure S1) were considered for genome analysis. Supplementary Table S1 summarizes accession number and general features of these genomes, in Supplementary Table S2 the accession for all 240 are listed. Sequences of 16S rRNA gene were obtained using barrnap, and targeted extraction and orientation verified with Metaxa2. Fragmented short ribosomal gene sequences < 700 bp were filtered out using an *ad hoc* Python script. In addition, we included the 16S rRNA gene sequences of isolates considered in this study for which a genome sequence is not available (*n* = 240). Sequences were first aligned with MAFFT, then trimmed with TRIMal and a refined alignment was performed using MUSCLE. Finally, we used ModelTest-NG to identify the best model for the construction of the Maximum-Likelihood tree in RAxML using the Transfer Bootstrap method for branch support. The genome of *Microbacterium* sp. 1.5R (CP018151) was sequenced and assembled as described in Corretto et al. (2015).

**FIGURE 1:**
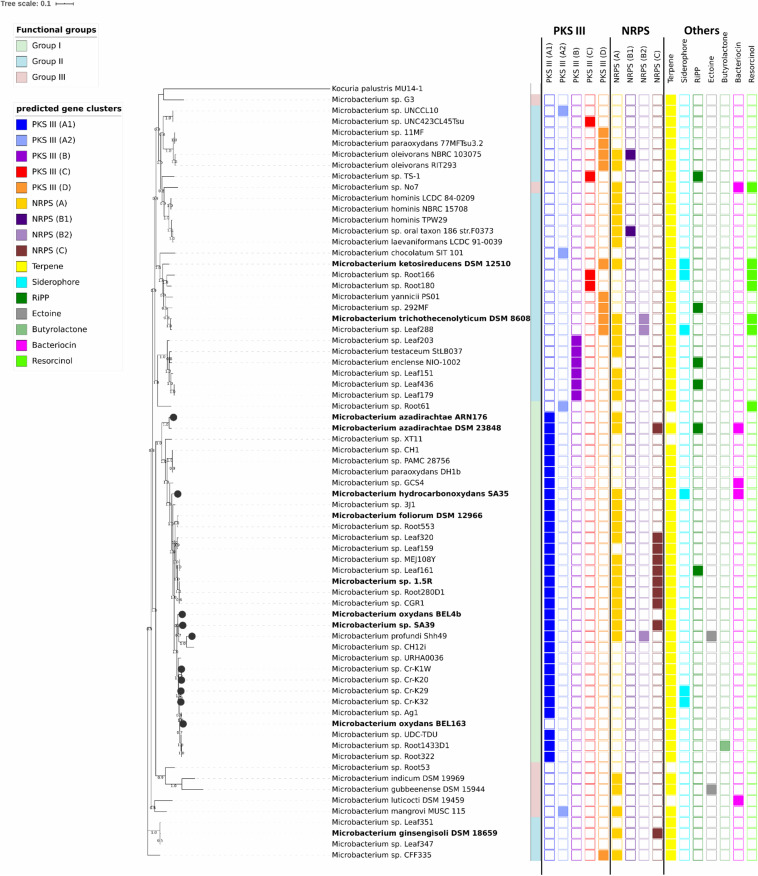
Maximum likelihood phylogenetic tree based on 38 concatenated unique marker genes identified using Phylosift (Darling et al., 2014). *Kocuria palustris* MU14/1 (CP012507) was used as outgroup. Bootstrap values > 50% are shown at branch points. Strains belonging to phylogenetic groups I, II and III are highlighted in green, blue, and red, respectively. Isolates in bold were tested in this study. Isolates marked with a black circle were isolated from heavy metal contaminated sites. The first column on the right side of the tree shows the different types of PKS gene clusters; whereas the second and third columns show the different types of NRPS gene clusters. The bars indicate the other predicted secondary metabolite gene clusters identified with antiSMASH 5.0.

Phylogenetic analysis was carried out with the following genes: (i) 16S rRNA gene sequences (Supplementary Figure S1 and Supplementary Table S2) and (ii) 38 unique marker genes identified using Phylosift v.1.0.1 (marker database v.4) (Darling et al., 2014) (Figure 1 and Supplementary Tables S2, S3) and (iii) core and accessory genes selected in pan-genome analysis (trees not shown). Sequences were aligned with MUSCLE v.3.8.31 (Edgar, 2004). Poorly aligned positions were removed using Gblocks v.0.91b with default parameters (Castresana, 2000), except for the 16S rRNA gene alignment. Maximum Likelihood trees were calculated in RAxML-NG v.0.8.0 (Kozlov et al., 2019) using the Transfer Bootstrap method for branch support (Lemoine et al., 2018) and according to the best-fit models selected with ModelTest-NG v.0.1.5 (Darriba et al., 2019). Tree were visualized using iTOL v4 (Letunic and Bork, 2019).

### Pan-Genome Construction

All genomes were re-annotated in Prokka v.1.13.3 (Seemann, 2014). The pan-genome was calculated with Roary v.3.11.2 (Page et al., 2015). We used default parameters and changed only the minimum blastp percentage identity to 80%. We divided the *Microbacterium* pan-genome in the following categories as calculated in Roary (Page et al., 2015): core (gene families present in 99–100% of the genomes, 69–70 genomes); soft-core (gene families present in 95–99% of the genomes, 67–69 genomes); shell (gene families present in 15–95% of the genomes, 11–67 genomes); cloud (gene families present in 0–15% of the genomes, 1–11 genomes).

### Functional Diversity Analysis

**FIGURE 2:**
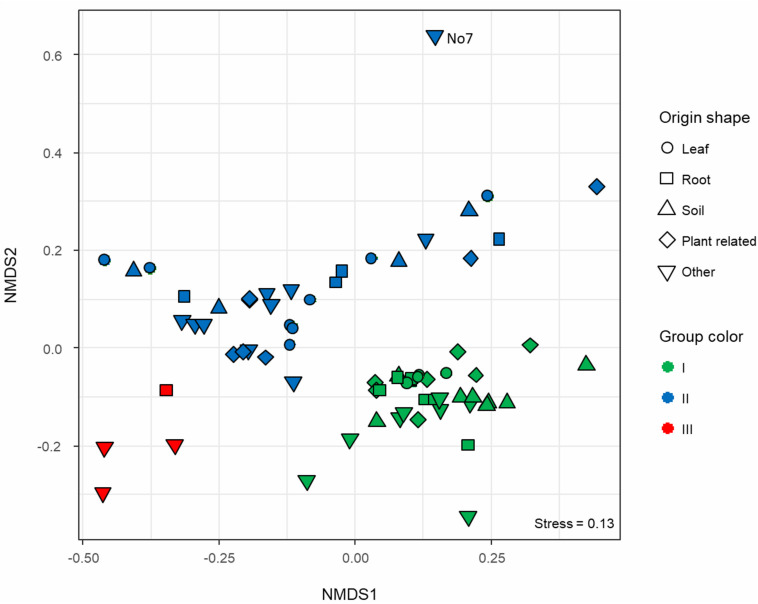
Non-metric multidimensional scaling (NMDS) ordination based on Bray–Curtis dissimilarity calculated on a contingency table where functionally annotated orthologous groups (eggNOG) were counted across all *Microbacterium* spp. genomes. Samples are displayed in different shapes according to the isolation origin, whereas the colors represent the functional group affiliation (green, blue, and red) as described in Figure 1.

Protein FASTA files of CDS sequences parsed and translated by Prokka (Seemann, 2014) were imported in a locally installed EggNOG v.1.0.3 mapper running in server mode (Huerta-Cepas et al., 2016). The functional annotation was carried out using the whole database based on bacterial orthologous groups (OG). The annotation outputs of each genome were then concatenated and formatted using an *ad hoc* shell script and imported in R for further statistical analysis. Unassigned proteins were removed. A contingency table was built counting the occurrences of each OG, resulting in a matrix were genomes are the samples and GOs the featuring variables. An exploratory multivariate analysis was conducted using a non-metric multidimensional scaling (NMDS) based on Bray–Curtis dissimilarity (vegan: Community Ecology Package. R package version 2.4-6^1^) and a stress value reported (Figure 2). A statistical analysis was then performed on the same dissimilarity matrix to assess the significance of the origin (isolation) and the phylogenetic group via permutational multivariate analysis of variance (PERMANOVA; Anderson, 2001). A confirmatory analysis of deviance was then computed for a multivariate Generalized Linear Model (GLM) based on a negative binomial regression, with 999 iterations (mvabund: Statistical Methods for Analysing Multivariate Abundance Data. R package version 3.13.1^2^).

### Prediction of Secondary Metabolite Gene Clusters

The Genbank files obtained from the Prokka annotation (Seemann, 2014) were uploaded in antiSMASH 5.0 (Blin et al., 2019). The comparison of the terpenoid clusters was restricted to the *Microbacterium* strains that have a sequenced genome and were tested in this study, so that we could directly determine the color of the colonies.

### Tested Isolates

A total of 29 bacteria belonging to the *Microbacterium* genus were obtained from different culture collections in order to assess their plant growth-promoting features, resistance to heavy metals and the ability to influence metal mobility in contaminated soil (Table 1). Experimental procedures are described in the next paragraphs. Strains SA5b, SA35, and SA39 were kindly provided by the Instituto de Investigaciones Agrobiológicas de Galicia (IIAG), Consejo Superior de Investigaciones Científicas (CSIC), Santiago de Compostela, Spain. Strains BEL125b, BEL156, and BEL163 were kindly provided by the Hasselt University, Centre for Environmental Sciences, Diepenbeek, Belgium. The DSM strains were obtained from the Leibniz Institute DSMZ-German Collection of Microorganisms and Cell Cultures. The rest of the strains are part of the internal collection of the Austrian Institute of Technology. The corresponding papers on already published strains are provided in Table 1.

**TABLE 1:** Phylogenetic affiliation and features of 29 *Microbacterium* isolates tested in this study.

Isolation source (reference)	Isolate	Identification [BioSample/Acc. Num.]	PGP traits				MIC (mM)		
			IAA	ACC	SolP	Sider	Zn	Pb	Cd
**Heavy metal contaminated site**									
Soil (Austria) (Corretto et al., 2015)	ARN176	*M. azadirachtae* [SAMN03266145]	+	+	+	−	14	6	−
*Alyssum serpyllifolium* ssp. *Lusitanicum* rhizosphere (Portugal) (Corretto et al., 2015)	SA35	*M. hydrocarbonoxydans* [SAMN03266143]	+	+	+	−	12	6	−
*Brassica napus* rhizosphere (Belgium) (Corretto et al., 2015)	BEL4b	*M. oxydans* [SAMN03266142]	−	+	−	+	8	2	1
*Salix viminalis* roots (Belgium)	BEL156	*M. oxydans*[KY091266]	+	−	+	−	12	6	2
*Salix viminalis* roots (Belgium) (Corretto et al., 2015)	BEL163	*M. oxydans* [SAMN03256326]	+	−	+	−	12	6	2
*Brassica napus* rhizosphere (Belgium)	BEL125bM	*M. phyllosphaerae* [KY091267]	−	+	−	+	6	6	1
*Salix caprea* leaves/green branches (Austria) (Kuffner et al., 2010)	EX104	*Microbacterium* sp. [GQ342547]	+	+	+	+	14	6	−
*Salix caprea* leaves/green branches (Austria) (Kuffner et al., 2010)	EX72	*Microbacterium* sp. [GQ342563]	+	−	+	−	14	10	6
*Alyssum serpyllifolium* ssp. *Lusitanicum* rhizosphere (Portugal)	SA5b	*Microbacterium* sp. [HF570056]	+	+	+	−	12	6	−
*Alyssum serpyllifolium* ssp. *Lusitanicum* rhizosphere (Portugal) (Corretto et al., 2015)	SA39	*Microbacterium* sp. [SAMN03266144]	+	+	−	−	2	2	−
**Non-heavy metal contaminated site**									
*Cynodon dactylon* shoots (Austria)	8.11S	*M. arborescens* [KY091268]	−	+	+	−	1	1	−
Common ragweed stems (Austria)	444	*M. arborescens* [KY091269]	−	+	+	−	−	−	−
Rhizoplane of neem *Azadirachta indica* seedlings (India) (Madhaiyan et al., 2010)	DSM 23848	*M. azadirachtae* [SAMN03256324]	+	+	+	−	14	8	0.5
Phyllosphere of grasses (Germany) (Behrendt et al., 2001)	DSM 12966	*M. foliorum* [SAMN03256325]	−	+	+	+	6	2	−
Soil of ginseng field (South Korea) (Park et al., 2008)	DSM 18659	*M. ginsengisoli* [SAMN03266139]	+	−	+	+	14	10	6
Common ragweed stems (Austria)	138	*M. hydrocarbonoxydans* [KY091270]	−	−	−	+	2	2	−
Soil (Takeuchi and Hatano, 1998)	DSM 12510	*M. ketosireducens* [SAMN03266140]	−	+	−	+	1	2	−
Common ragweed roots (Austria)	280	*M. oleivorans* [KY091271]	+	+	+	+	2	2	−
Phyllosphere of grasses (Germany) (Behrendt et al., 2001)	DSM 13468	*M. phyllosphaerae* [AJ277840]	−	+	−	+	10	2	−
Common ragweed rhizosphere (Austria)	228	*M. pumilum* [KY091272]	−	+	−	−	1	10	−
Soil (Yokota et al., 1993)	DSM 8608	*M. trichothecenolyticum* [SAMN03266141]	+	+	+	−	1	2	−
Potato roots (Peru)	P1-3	*M. yannicii* [KY091273]	+	+	−	+	6	2	−
*Linum austriacum* ssp. *austriacum* roots (Austria)	1.5R	*Microbacterium* sp. [SAMN05992342]	−	+	−	+	6	2	−
Common ragweed roots (Austria)	8	*Microbacterium* sp. [KY091274]	−	−	+	−	2	1	−
*Cynodon dactylon* shoots (Austria)	8.4Sa	*Microbacterium* sp. [KY091275]	−	+	+	−	2	1	−
Common ragweed rhizosphere (Austria)	507	*Microbacterium* sp. [KY091276]	+	+	−	+	−	−	−
Common ragweed rhizosphere (Austria)	625	*Microbacterium* sp. [KY091277]	+	+	−	−	−	−	−
*Glycine max* nodule (Austria)	KO1	*Microbacterium* sp. [KY091278]	+	+	−	−	2	−	−
*Glycine max* nodule (Austria)	KO2	*Microbacterium* sp. [KY091279]	+	+	+	−	−	−	−

**Strains with a sequenced genome are linked to their BioSample numbers; whereas the others were identified by sequence analysis of the 16S rRNA gene (see Supplementary Figure S1 for methods). The corresponding papers of already published strains are provided in the “isolation source” column. Plant growth-promoting (PGP) traits: IAA, production of indole-3-acetic acid; ACC, aminocyclopropane-1-carboxylate deaminase activity; Sol P, phosphate solubilization; Sider, production of siderophores. MIC, minimal inhibitory concentration*.*

Out of the 29 isolates, 11 strains have a sequenced genome. All isolates were plant- or soil-associated and were isolated from contaminated sites (10 isolates) and from non-contaminated sites (19 isolates) (Table 1). Cells were routinely grown at 27°C in Landy medium (20 g l^–1^ glucose, 5 g l^–1^ glutamate, 0.25 g l^–1^ MgSO_4_, 0.25 g l^–1^ KCl, 0.5 g l^–1^ KH_2_PO_4_, 150 μg l^–1^ FeSO_4_, 5 mg l^–1^ MnSO_4_, 160 μg l^–1^ CuSO_4_, 1 g l^–1^ yeast extract, pH 7.2) (Landy et al., 1948).

### *In vitro* Tests

Resistance to heavy metals was tested on solid Landy medium supplemented with the following metals: zinc sulfate (1–14 mM), lead nitrate (1–10 mM) and cadmium nitrate (0.5–6 mM). Growth was assessed after 7 days of incubation at 27°C.

In addition, the isolates were tested for four common plant growth promotion activities: production of siderophores and auxins, phosphate mobilization and ACC deaminase activity. Siderophore production was analyzed using the modified CAS agar plate assay described in Milagres et al. (1999). Briefly, the agar plates were divided in two parts: one half containing solid Landy (without the addition of 150 μg l^–1^ FeSO_4_) as growth medium and the other half containing CAS agar prepared according to Schwyn and Neilands (1997). Plates were incubated for 5 days at 27°C. The ability to solubilize phosphate was tested on Pikovskaya agar plates (Pikovskaya, 1948): positive isolates produced a halo-zone around the colony after 5 days at 27°C. Production of indole acetic acid (IAA) was detected with the colorimetric assay described in Loper and Schroth (1986). Bacteria were inoculated in 5 ml Landy medium with the addition of 500 μg ml^–1^of tryptophan and were incubated at 27°C for 72 h at 200 rpm. Cells were removed by centrifugation (13,000 rpm, 10 min). To 2 ml of the supernatant, 40 μl orthophosphoric acid (85%) and 4 ml Salkowski reagent (50 ml perchloric acid 35%, 1 ml FeCl_3_ 0.5 M) were added. After 10 min of incubation in the dark, absorbance at 530 nm was measured. Indole-3-acetic acid was used as standard for quantification. ACC deaminase activity was tested on a minimal medium containing 0.7 g l^–1^ ACC as sole nitrogen source (Brown and Dilworth, 1975). Minimal medium without nitrogen was used as negative control, whereas the positive control plates contained 0.7 g l^–1^ NH_4_Cl. Plates were incubated at 27°C for 2 weeks.

### Detection of Genes Associated to Plant Growth Promotion Activity, Metal Resistance/Homeostasis and Iron Uptake Strategies

For this analysis, out of the 29 isolated, we selected the 10 *Microbacterium* genomes presented in Corretto et al. (2015) and *Microbacterium* sp. 1.5R (CP018151). These bacteria were selected for genome sequencing based on the following criteria: resistance to different concentrations of heavy metals (zinc, lead, and cadmium); different isolation sources. Five of the strains were isolated from contaminated sites and six from not-contaminated sites across Europe. These genomes were further annotated in RAST (Overbeek et al., 2014). The WebMGA server (Wu et al., 2011) was used to assign COG functional categories. Odds ratio (OR) of the different x categories (heavy metal relates genes, metallo-sensing regulators, arsR, genes belonging to the COG P category for inorganic ion transport and metabolism) were calculated as follows: OR = (*A*/*B*)/(*C*/*D*) with *A* being the number of genes in the genome(s) of interest assigned to a category *x*; *B* the number of genes in the genome(s) of interest assigned to all COG categories; *C* the number of genes in all the sequenced genomes assigned to a category *x*; *D* the number of genes in all the sequenced genomes assigned to all COG categories. Each contingency table was tested for significance using the one-tailed Fisher exact test (*P* ≤ 0.05). as described in Hemme et al. (2016).

### Heavy Metal Mobilization Assay

A previous study (Kuffner et al., 2010) showed that bacterial exudates of *Microbacterium* spp. cultures mobilize metals in contaminated soils, particularly when collected during the stationary phase. Therefore, bacteria were grown in 50 ml of Landy medium at 27°C and 200 rpm until the stationary phase. Cells were removed by centrifugation (4,700 rpm, 20 min, 4°C) and the supernatants were filtrated through 0.2 μm filters (Millipore). Filtrates were stored at −20°C and pH values were measured prior starting the extraction assay. For each strain two cultures were prepared and from each culture three aliquots were analyzed as follows. Five ml of filtrates were shaken with 1 g of Zn, Cd, and Pb-contaminated soil for 2 h at room temperature. Soil characteristics can be found in Supplementary Table S11. As negative control, 1 g of soil was shaken with 5 ml of sterile Landy medium, which was adjusted to pH values between 6.5 and 3.7 to mimic the medium acidification caused by bacterial growth in some cultures. Soil particles were removed by centrifugation (7,000 rpm, 5 min), the supernatants were filtered through 0.45 μm filters and acidified to 2% HNO_3_ (p.a. grade, Sigma-Aldrich, Vienna, Austria). Concentration of Zn, Cd, Pb, Fe, Cu, and Mn were quantified by inductively coupled plasma mass spectrometry (Elan DRCe 9000, PerkinElmer).

## Results

### Phylogenetic and Functional Diversity Analysis of 70 *Microbacterium* Genomes

The analyzed genomes represent at least 20 different *Microbacterium* species that were isolated from a broad range of habitats, mainly plants and soils (Supplementary Table S1). We found an average genome size of more than 3.5 Mb, a GC% content of about 70% and more than 3,300 coding sequences per genome.

To elucidate the evolutionary relationships between the selected organisms, we performed a phylogenetic analysis using different sets of genes: the 16S rRNA gene as conventional marker (Supplementary Figure S1) and 38 unique marker genes (Figure 1 and Supplementary Table S3) that were identified by Phylosift. The 16S rRNA gene tree built with 240 genomes differs from the one based on the 38 unique marker genes and does not ensure sufficient phylogenetic resolution for a number of species. For instance, the strains showing high similarity to *Microbacterium oxydans*, *Microbacterium hydrocarbonoxydans*, *Microbacterium phyllosphaerae* and *Microbacterium foliorum* are grouped on one single branch (Supplementary Figure S1).

To characterize the functional potential of the selected *Microbacterium* strains, each genome was annotated using the eggNOG database. Functional diversity analysis shows that there are three main distinct operational functional groups. As exception, *Microbacterium* sp. No. 7 seems to be quite distinct from these groups (Figure 2), even though in the phylogenetic tree is positioned next to organisms belonging to the group II (Figure 1). Also, *Microbacterium* sp. G3 from group III seems to be functionally closer to group I, even though in the phylogenetic tree is located among organisms belonging to group II. Statistical analysis (PERMANOVA; Anderson, 2001) suggests that the taxonomic affiliation (pseudo-*F* = 10.41, *R*^2^ = 0.23, *p* < 0.001) has a higher influence than the isolation source (pseudo-*F* = 1.76, *R*^2^ = 0.08, *p* < 0.001) on the formation of the different functional clusters. Since the dispersion of variances resulted in not homoscedastic distribution for taxonomic affiliation, a confirmatory multivariate GLM followed by Likelihood-ratio test was applied. The model was validated with a residual plot and confirmed previous results (LRT_tax.aff_ = 39920.88, LRT_iso.sor_ = 34704.79, *p* = 0.001).

To further investigate the selected *Microbacterium* genomes, we performed a pan-genome analysis. The *Microbacterium* pan-genome has a total of 83,207 gene orthologs: 331 are shared between the core (165) and soft-core (166) genome; while 3,220 and 79,656 clusters form the shell and the cloud genome, respectively. Trees calculated with core and accessory genes show the presence of three main groups, which overlap with the functional groups described above. All COG categories are equally distributed among the analyzed *Microbacterium* genomes (data not shown). However, most of the core genes have housekeeping functions and belong to the COG categories of translation, ribosomal structure and biogenesis (J) or amino acid/nucleotide transport and metabolism (E/F). On the other hand, unique and accessory genes (cloud) have unknown function (S) or fall within more specific COG categories like carbohydrate transport and metabolism (G) and inorganic ion transport and metabolism (P).

Based on the nature (open/closed) and the size of the pan-genome, one can speculate on the lifestyle of an organism and its ability to acquire exogenous DNA, therefore gaining new advantageous functions (Rouli et al., 2015). Supplementary Figure S2 shows the effect on the total size of the pan-genome every time a new genome is added. The curve increases at each genome addition, but a plateau is not reached with the number of investigated genomes. This observation indicates that the *Microbacterium* pan-genome is open.

### Secondary Metabolite Potential of *Microbacterium* Genomes

The analysis of secondary metabolite gene clusters with antiSMASH 5.0 (Blin et al., 2019) revealed the presence of several clusters for saccharides or fatty acids production (data not shown). These clusters showed low homology compared to the already characterized ones deposited in the MIBiG database^3^. In addition, we could detect and identify gene clusters for the production of terpenoids (in 96% of the analyzed genomes), type III polyketide synthase (PKS) genes (79%), non-ribosomal peptide synthetase (NRPS) genes (64%) and rarer clusters (<10%) involved in the production of siderophores, resorcinol, bacteriocins, ectoine, butyrolactone and ribosomally synthesized post-translationally modified peptides (RiPP) (Figure 1 and Supplementary Table S3).

#### Siderophore Gene Clusters: NRPS-Dependent and –Independent Pathways

Six *Microbacterium* genomes contain a cluster showing similarity to the one for the synthesis of desferrioxamine. All four biosynthetic genes (*desABCD*) are present, but not the related transporters (*desEF*) (Supplementary Table S5). Only few genes showed low amino acid identity (30–40%) with *desF* and *cdtB*, which is part of the ferric-siderophore transporter gene cluster (*cdtABC*) in *Streptomyces* species (Patel et al., 2010; Terrafria et al., 2011).

Several siderophores are synthesized by NRPS–dependent pathways. The NRPSs described here were identified in antiSMASH under the categories “nrps” and “other.” Within the “nrps” category, only four clusters were detected among the selected *Microbacterium* genomes (Supplementary Table S6). The clusters present in the genomes of *Microbacterium yannicii* PS01 and *Microbacterium azadirachtae* DSM 23848 contain genes harboring the characteristic NRPS domains: adenylation (A), peptidyl carrier protein (PCP) and condensation (C) domains. These genes did not show similarity to previously characterized NRPS gene clusters, impairing any prediction on the produced compounds. On the other hand, the clusters present in *Microbacterium* sp. No. 7 and *Microbacterium gubbeenense* DSM 15944 seem to be responsible for the synthesis of siderophores similar to enterobactin and griseobactin, respectively (Crosa and Walsh, 2002; Patzer and Braun, 2010) (Supplementary Table S6).

The antiSMASH category “other” is meant for a “cluster containing a secondary metabolite-related protein that does not fit into any other category.” However, in the case of the *Microbacterium* genomes, all the detected clusters contained NRPS genes. We divided these clusters in three types (A, B1/2, C) based on the predicted substrate used by the A domain of the NRPS gene (Figure 1 and Supplementary S6). Type A NRPSs use glycine, alanine or a hydrophilic compound as substrate and possess three essential domains: an adenylation (A) domain of siderophore synthetizing NRPSs (SidN3_like), a peptidyl carrier (PCP) domain and a terminal NRPS associated domain having acyltransferase activity and lipase-esterase activity only in the case of *Microbacterium* sp. No. 7 (Figure 1). In these clusters, right next to the NRPS gene, we could always identify an aminopeptidase N, which can function as a thioesterase domain (TE) releasing the product by hydrolysis. NRPS genes belonging to the cluster type B1/2 harbor the similar domains but can bind different substrates: polar (asn, gln, asp, glu, and 2-amino-adipate) (type B1) or hydrophobic/aliphatic amino acids (type B2) (Figure 1). The cluster type C NRPSs contain only the A domain (adenylate forming domain, AFD, class I superfamily). Apart from the NRPS genes identified in antiSMASH, we could find a group of genes belonging to the NRPS cluster type C and annotated as non-ribosomal protein synthetases, which are shorter than the ones described above (400–500 aa). These NRPSs have only a C domain of unknown function and are flanked by hydrolases (Figure 1 and Supplementary Figure S6).

The NRPS clusters are randomly distributed along the whole phylogenetic tree (Figure 1), suggesting they might be the result of horizontal gene transfers.

#### Type III Polyketide Synthases (PKS)

A total of 79% of the analyzed *Microbacterium* genomes harbor a type III PKS with a characteristic chalcone and stilbene synthase domain (Supplementary Table S4). We could define four different types of PKS clusters based on the genes flanking the *pks11* or *pks18* gene: types A (1/2), B, C and D (Figure 1 and Supplementary Figure S7). All the clusters harbor a long-chain fatty acid-CoA ligase (FadD15), that probably provides long-chain fatty acid starter units. The most common one is the type A, which can be divided in two subgroups based on the presence of different long-chain fatty acid-CoA ligase (FadD15). The ligases of PKS cluster types B, C, and D form a separate group and are positioned far away from the cluster or they are not included within the cluster due the genome fragmentation (Supplementary Figure S8). Organisms of PKS cluster type B contain a polyketide cyclase and separate clearly from the cluster type C, harboring genes with PKS/NRPS domains, and type D (Figure 1).

Overall, these results suggest that *Microbacterium* species might produce at least four different PKS types which are distributed in groups that largely correspond to the three main operational functional groups (Figure 1).

#### C_50_ Carotenoids Gene Clusters

Among the identified secondary metabolite gene clusters, the terpenoid cluster was the most commonly found. These compounds confer a yellow color to the colonies, whereas organisms lacking this cluster are usually white (Supplementary Table S3) (Qian et al., 2007; Vaz-Moreira et al., 2008; Corretto et al., 2016). The synthesis of these pigments can be influenced by the growth conditions like the presence/absence of light (Trutko et al., 2005; Meddeb-Mouelhi et al., 2016). In this study, the strains harboring the terpenoid cluster had pigmented colonies despite being grown in the dark.

The organization of the genes within the analyzed clusters resembles the one of C_50_ carotenoids like decaprenoxanthin, sarcinaxanthin or C.p. 450 (Heider et al., 2014), of which both the biosynthetic pathway and the chemical structure have already been characterized in *Corynebacterium glutamicum* ATCC 13032 (Heider et al., 2012), *Micrococcus luteus* NCTC 2665 (Netzer et al., 2010) and *Dietzia* sp. CQ4 (Tao et al., 2007), respectively. We performed a phylogenetic analysis using the main biosynthetic genes: lycopene elongase, geranylgeranyl-pyrophosphate synthase (GGPS), two cyclases and glycosyl transferase (Supplementary Figures S3, S4). The topology of the trees showed that the genes of *Microbacterium* species cluster in two main groups A, B (with the exception of *Microbacterium hydrocarbonoxydans* SA35 and *M. azadirachtae* DSM 23848) and separate well from the previously characterized genes of *C. glutamicum* ATCC 13032, *Dietzia* sp. CQ4 and *M. luteus* NCTC 2665. Isolates from both groups produce a methanol dissolvable yellow pigment, extractable from the water phase with chloroform only after hydrolysis. The pigment contains a nona-ene chromophore, pointing to a structure similar to glycosylated sarcinaxanthin or decaprenoxanthin (Supplementary Figure S5). Potential differences in the exact pigment structure due to differentiated biosynthetic genes require further investigation.

A specific glycosyl transferase catalyzes the addition of an activated sugar molecule via glycosylation. This gene can either flank the terpenoid cluster, as in *M. luteus* NCTC 2665 and *Dietzia* sp. CQ4, or can be separated by several kilobases as in *C. glutamicum* ATCC 13032 (Netzer et al., 2010). Blasting the glycosyl transferase of *M. luteus* NCTC 2665 against the analyzed *Microbacterium* genomes, we could identify glycosyl transferases that are located far away from the terpenoid cluster, but are possibly involved in the carotenoid glycosylation process (Supplementary Figure S4).

#### Other Secondary Metabolite Gene Clusters

Ribosomally synthesized post-translationally modified peptides (RiPP) are another huge group of structurally and functionally different metabolites (Ortega and van der Donk, 2016). Among the *Microbacterium* genomes, we could find only six gene clusters for RiPPs such as lantipeptides (*Microbacterium* sp. Leaf436 and TS-1), lassopeptides (*Microbacterium* sp. 292MF), ladderanes (*Microbacterium enclense* NIO-1002), thiopeptides (*Microbacterium* sp. Leaf161) and linaridin (*M. azadirachtae* DSM 23848).

Interestingly, only *Microbacterium profundi* Shh49, isolated from a deep-sea sediment, and *Microbacterium gubbeenenense* DSM 15944, isolated from cheese surface, possess a cluster for ectoine, which is an osmoprotectant widespread among halophilic and halotolerant microorganisms growing in saline environments (Reshetnikov et al., 2011).

### Plant Growth Promoting Activities

Besides exploring the genetic potential of *Microbacterium* species, our next goal consisted in the functional characterization of selected strains by testing their ability to promote plant growth. For this purpose, we collected 29 *Microbacterium* strains isolated from contaminated and non-contaminated European sites, of which 11 have a sequenced genome (Table 1).

First, we screened the 29 isolates for the most prominent plant growth-promoting (PGP) traits such as aminocyclopropane-1-carboxylate (ACC) deaminase activity, phosphate solubilization, production of siderophores and indole-3-acetic acid (IAA). A high percentage (79%) of the tested *Microbacterium* isolates showed ACC deaminase activity (Table 1). However, in the 11 sequenced genomes, we were not able to identify annotated ACC deaminase genes.

More than half of the isolates (59%) could produce IAA and solubilize phosphate (Table 1). All the analyzed genomes, except *M. oxydans* BEL163, contain the genes for the synthesis of tryptophan, the IAA main precursor, as well as genes necessary for its conversion to IAA through the tryptamine pathway (Spaepen and Vanderleyden, 2011) (Supplementary Table S7).

Within the RAST subcategory dedicated to phosphate metabolism, several genes were found in all genomes (Supplementary Table S8). This support the fact that 59% of the tested isolates were able to solubilize phosphate *in vitro*. Among the detected genes, many encode phosphatases for the utilization of organic phosphorus and for transporters and regulatory elements belonging to the PHO regulon, which controls the use of inorganic phosphate (Pi) (Baek and Lee, 2006; Santos-Beneit, 2015).

Microbes adopt different strategies for iron uptake and homeostasis. Among the tested isolates, 41% were positive in the CAS assay for the detection of siderophores. However, it has to be mentioned, that not all types of siderophores are detected by the CAS assay (Schwyn and Neilands, 1997; Milagres et al., 1999). The RAST subsystem dedicated to “iron acquisition and metabolism” gives an overview on the different mechanisms and compounds involved (Supplementary Table S9). We detected two putative siderophore biosynthetic genes and confirmed the presence of defererrioxamine biosynthetic gene clusters. Despite the small number of siderophore biosynthetic genes detected, the 11 analyzed genomes contain several transporters for iron-siderophore complexes (unspecified siderophore interacting proteins, petrobactin- or aerobactin-like siderophore transporters) as well as for ferrous iron (low pH induced EfeUOB transporters, homologs to *Streptococcus* Pit iron transporters).

### Interaction With Heavy Metals

Using the same 29 *Microbacterium* strains, we investigated other key features for the selection of strains employed in phytoextraction technologies. These include their resistance to heavy metals and their ability to change the metal mobility in contaminated soils. The investigation of these phenomena could help to understand the mechanisms that lead to adaptation to heavy metal-rich environments.

**FIGURE 3:**
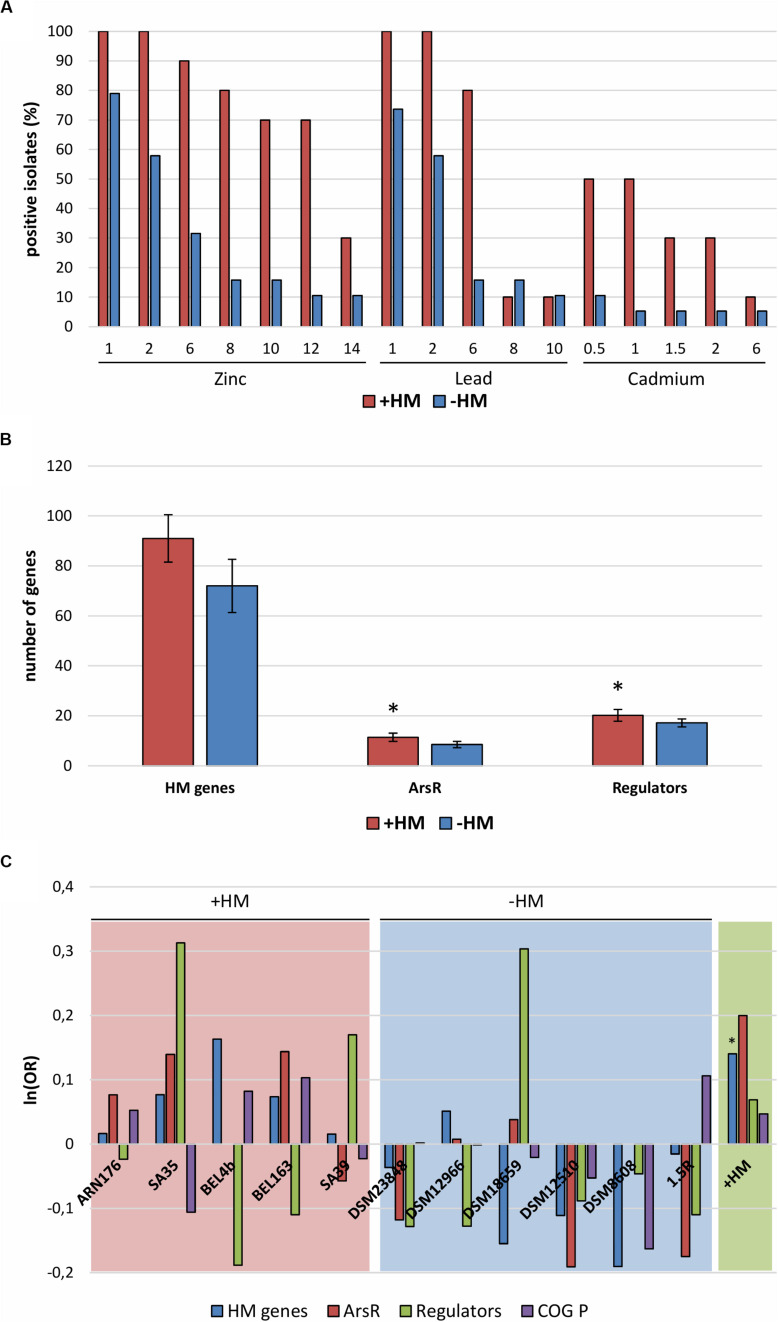
**(A)** Heavy metal resistance test: zinc sulfate (1–14 mM), lead nitrate (1–10 mM), and cadmium nitrate (0.5–6 mM). **(B)** Total number of genes related to heavy metal resistance and homeostasis (HM genes), of metallo-sensing regulators (regulators) and ArsR regulator family. Error bars show standard deviation. Asterisks indicate significantly different values of +HM compared to –HM (*p* < 0.05). **(C)** Odds ratio (OR) of genes belonging to single isolates compared to all sequenced bacteria (red and blue panels) and isolates from heavy metal contaminated sites compared from non-contaminated sites (green panel). Asterisks indicate significant deviation from the null hypothesis [ln(OR) = 0] at the 95% confidence level by one-tailed Fisher exact test. Gene categories: HM genes (blue), genes related to heavy metal resistance and homeostasis; ArsR (red), regulators belonging to the ArsR family; Regulators (green), metallo-sensing regulators; COG P (purple), genes assigned to the COG P category (Inorganic ion transport and metabolism). +HM, bacteria isolated from heavy metal contaminated sites (red); –HM, bacteria isolated from heavy metal non-contaminated sites (blue).

As expected, the number of resistant isolates decreased with the increase of metal concentration and its toxicity (Zn < Pb < Cd) (Figure 3A). Generally, *Microbacterium* isolated from metal contaminated sites were on average more resistant than the ones isolated from non-contaminated sites. Interestingly, all 11 sequenced genomes harbor genes related to metal homeostasis (transporters, permeases, and efflux pumps) and various metallo-regulators (ArsR, MerR, CsoR, Fur, Zur, and DtxR/IdeR) (Supplementary Table S10). However, in isolates from contaminated sites, genes related to heavy metal homeostasis and metallo-sensing regulators like *arsR* seem to be more abundant (Figures 3B,C).

**FIGURE 4:**
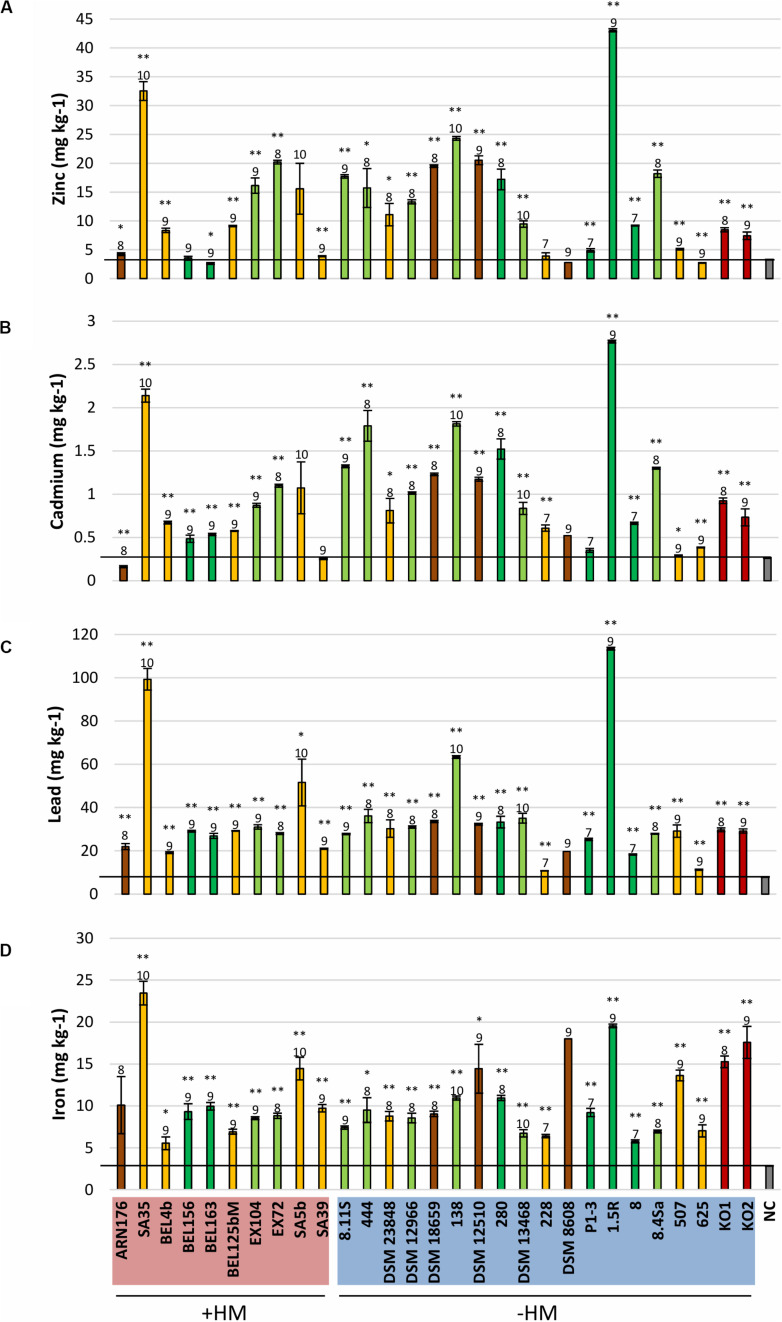
Mobilization of zinc **(A)**, cadmium **(B)**, lead **(C)**, and iron **(D)** from contaminated soil by bacterial exudates. The different colors of the diagram bars represent different isolation sources: shoots in light green; roots in dark green; rhizosphere in orange; soil in brown; nodules in dark red. NC, negative control (gray). +HM, bacteria isolated from heavy metal contaminated sites; –HM, bacteria isolated from non-contaminated sited. Error bars show standard errors of the mean (*n* = 6, except for NC *n* = 3 and for bacteria producing a polysaccharide matrix difficult to filtrate: *n* = 5 for EX104 and 280; *n* = 3 for 228 and K02; *n* = 2 for K01; *n* = 1 for DSM 8608). Samples with extraction values differing significantly from the control (NC) are labeled with ^∗^(*p* < 0.05) and ^∗∗^(*p* < 0.01). The order of magnitude of CFU/mL prior to filtration is indicated above the diagram bars. The graphs representing the mobilization of copper and manganese can be found in the Supplementary Material, Supplementary Figure S9.

The ability of enhancing the mobility of metals such as iron, zinc, cadmium, lead, and manganese seems to be widespread among the tested isolates, irrespective of their isolation source (Figure 4 and Supplementary Figure S9). Copper was the only metal that was immobilized by all isolates except *Microbacterium* sp. 625 (Supplementary Figure S9). The mobilization effect was partly caused by a decrease in pH (from 7.2 to 4.5) and partly due to other processes, such as the secretion of metabolites during the growth in Landy medium (Supplementary Figure S10).

Such mobilization experiments provide information about the mobilization ability of the selected organisms. In order to verify if the same metabolites are produced not only from the substrates present in a lab growth medium, but also from the substrates available in a contaminated soil, we selected three *Microbacterium* strains with high (*M. hydrocarbonoxydans* SA35, *Microbacterium* sp. 1.5R) and low (*M. azadirachtae* ARN176) metal mobilization efficiency (see section “Materials and Methods” for the greenhouse assays can be found in the Supplementary Material). The inoculation of *Brassica napus* and *Noccacea caerulescens* grown on a contaminated soil did not cause an increase in the biomass production (Supplementary Figure S11). Surprisingly, the two best mobilizers, *M. hydrocarbonoxydans* SA35 and *Microbacterium* sp. 1.5R could decrease the cadmium concentration in *B. napus* shoots, but had no significant effect on the other metals. The weak mobilizer, *M. azadirachtae* ARN176 acted as an immobilizer causing a decrease in the concentration of zinc, cadmium, manganese, and copper in the shoots and manganese, cadmium and lead in the roots (Supplementary Figures S12, S13). In contrast, we observed no differences between the treated and untreated *N. caerulescens* plants (Supplementary Figure S12): only *Microbacterium* sp. 1.5R could increase the concentration of manganese and copper.

## Discussion

*Microbacterium* species are Gram-positive bacteria, affiliated to the Actinobacteria phylum. In the last years, there has been an increased interest in *Microbacterium* strains isolated from heavy metal contaminated sites. Understanding their relationship with metals, the associated plants and exploring their genomes will provide insights on how these microbes adapted to such environments and how to exploit them for soil remediation processes. For this purpose, we analyzed 70 *Microbacterium* genomes and assessed the features of 29 *Microbacterium* strains as candidate organisms to speed up phytoremediation processes.

Previous phylogenetic studies showed that *M. oxydans*, *M. hydrocarbonoxydans*, *M. phyllosphaerae*, and *M. foliorum* are closely related species located on neighboring tree branches (Behrendt et al., 2001; Schippers et al., 2005). The Maximum Likelihood tree calculated with the 16S rRNA gene (Supplementary Figure S1) grouped these organisms in one big branch. By performing a multilocus sequence analysis, we could achieve a higher resolution resulting in a more robust phylogenetic analysis separating these species in different branches (Figure 1). The analyzed genomes can be grouped in three operational functional groups (Figure 2). Statistical analysis showed that taxonomy is the driving force defining these groups, whereas the isolation source has less influence.

The *Microbacterium* pan-genome is open. A wide and open pan-genome is usually associated with organisms living in a community with a high rate of horizontal gene transfer (Rouli et al., 2015). A total of 331 gene families are part of the core and soft-core of the pan-genome calculated with the 70 *Microbacterium* genomes. This extended core genome accounts for about 10% of the genes present in a typical *Microbacterium* genome (Table 2). The relatively small size of the extended core genome was obtained using a rather low blastp sequence identity threshold (80%), suggesting that the *Microbacterium* genus comprises species with high genetic diversity. Using 20 closely related strains and the An’vio pipeline, Learman et al. (2019) obtained a bigger core genome (26%), where the main COG categories were devoted to housekeeping functions as “Cellular processing and signaling” and “Information storage and processing.” As in this study, genes of the COG category “Inorganic ion transport and metabolism” or with unassigned function were mostly part of the accessory genome. They also registered a large genomic variability corresponding to different tolerance degrees to heavy metals and antibiotics (Learman et al., 2019). It is known that members of the *Microbacteriaceae* family have on average smaller core genomes compared to other Actinobacteria families like *Micrococcaceae*, *Cellulomonadaceae*, and *Streptomycetaceae* (Bai et al., 2015). In addition, the analyzed *Microbacterium* genomes have on average higher functional diversity compared to other members of the *Microbacteriaceae* family like *Agromyces*, *Curtobacterium*, and *Plantibacter* (data not shown). Taken together, *Microbacterium* genomes are characterized by a small number of conserved genes (core) covering basic functions (ribosomal proteins, elongation factors, transcriptional regulators, etc.) and by a high number of accessory genes having different sequences but similar or overlapping functions.

**TABLE 2:** *Microbacterium* pan-genome composition.

Pan-genome subgroup	Num. clusters of orthologous genes	Num. genes in subgroup	Num. genes/Num. genomes	Num. genes/average num. genes in *Microbacterium* genome (%)
Core	165	16369	233.84	9.76
Soft-core	166	6551	93.59	
Shell	3220	77078	1101.11	32.82
Cloud	79656	134880	1926.86	57.43
Total	83207	234878	3355.4	100

Compared to well-known Actinobacteria like *Streptomyces*, *Microbacterium* have smaller genomes (10 Mb vs. 3.5 Mb). Consequently, they host a lower number of secondary metabolite gene clusters (Jackson et al., 2019). Nonetheless, several secondary metabolite gene clusters were predicted by antiSMASH. They belong to different classes with NRPS, PKS III and terpenoids as the most common ones.

The presence of a desferrioxamine biosynthetic gene cluster, together with NRPS-like siderophore biosynthetic genes, suggest that *Microbacterium* species are able to synthesize siderophores via NRPS-dependent as well as -independent pathways. The NRPS genes are randomly distributed along the phylogenetic tree. This pattern suggests that these genes might be acquired via horizontal gene transfers. However, we have no information on the NRPS-siderophore type structures. In *Streptomyces* species, desferrioxamines have a role as regulatory or signaling molecules influencing cell growth and mediating ecological interactions with other bacteria and plants. The desferrioxamine biosynthetic pathway has been previously found in *Streptomyce*s species and related genera like *Salinispora* and *Agromyces* (Terrafria et al., 2011; Roberts et al., 2012; Corretto et al., 2016). In *Streptomyces coelicor* A3(2), the gene cluster consists of four biosynthetic genes (*desABCD*) and two transporter genes (*desEF*) with high specificity to desferrioxamine E. Some *Streptomyces* species have a second ferric-siderophore transporter gene cluster (*cdtABC*) that binds desferrioxamine B and other hydroxamate-containing siderophores (Patel et al., 2010; Terrafria et al., 2011). Here, we were able to identify the four biosynthetic genes (*desABCD*), but only few genes showed similarity to the transporters. Eto et al. (2013) showed that three *Microbacterium* isolates (*M. phyllosphaerae* KW016, *Microbacterium flavescens* KW111 and KW080) grew only in the presence of desferrioxamine B, but to our knowledge this is the first detailed report about desferrioxamine biosynthetic genes in *Microbacterium* species. Only few *Microbacterium* tested in this study were positive in the CAS test for iron solubilization. This result might be explained by the fact that many bacteria harbor transporters with a broader specificity range to exploit siderophores produced by other microbes (D’Onofrio et al., 2010; Terrafria et al., 2011; Mitter et al., 2013) or they might as well adopt other strategies for iron uptake such as acidification (Miethke et al., 2013; Mathew et al., 2014).

Type III PKS genes were first described in plants and later discovered in bacteria as well (Funa et al., 1999; Yu et al., 2012). These are homodimeric enzymes that iteratively catalyze condensation reactions to produce different polyketides like chalcones, pyrones and stilbenes (Shen, 2003; Yu et al., 2012). The identified PKS genes are annotated as “α-pyrone synthesis polyketide synthase-like Pks11 or 18.” Such genes have been previously characterized in other Gram-positive bacteria like *Streptomyces coelicolor* (Yu et al., 2012), *Mycobacterium tuberculosis* (Saxena et al., 2003) and *Bacillus subtilis* (Nakano et al., 2009). These studies showed that PKS11 and PKS18 usually accept long-chain aliphatic acid CoA-esters (C_6_–C_20_) as starter units rather than small-chain substrates to produce alkylpyrones and alkylresorcinols. The different types of PKS III clusters defined in our study roughly correspond to the phylogenetic distribution of the analyzed organisms. Thus, we can speculate that PKS III are vertically transmitted, whereas NRPS were acquired horizontally.

In nature, carotenoids are synthetized by plants, algae, fungi, and bacteria to carry out essential functions involved in nutrition, photosynthesis and protection against oxidative stress. In particular, C_50_ carotenoids have higher antioxidative properties compared to carotenoids with shorter hydrophobic backbones (C_25_, C_30_, and C_40_) and are known only from a small number of Gram-positive bacteria (Pfander, 1994; Heider et al., 2014). The presence of terpenoids clusters in *Microbacterium* strains has been recently documented (Han et al., 2016). However, there are only few studies describing the chemical structure of the produced carotenoids (Trutko et al., 2005; Godinho and Bhosle, 2008; Meddeb-Mouelhi et al., 2016). For instance, *Microbacterium* sp. LEMMJ01 produces a mix of carotenoids including neurosporene, which confer protection against UV-A and UV-B radiation (Reis-Mansur et al., 2019). Another key function of carotenoids is the stabilization of membranes. The presence of glycosylated groups at the molecule ends enhances the association of the carotenoids to the membrane, therefore increasing its rigidity and consequently the resistance to toxic compounds and osmotic stress (Britton, 2008). C_50_ carotenoids can be glycosylated through the addition of an activated sugar molecule catalyzed by a specific glycosyl transferase that has been characterized in *M. luteus* NCTC 2665 (Netzer et al., 2010). In the analyzed *Microbacterium* genomes, we could identify glycosyl transferases that are located far away from the terpenoid cluster, but are possibly involved in the carotenoid glycosylation process.

In heavy metal contaminated environments both bacteria and plants are highly sensitive to abiotic/biotic stresses. It is recognized that PGP bacteria can help the plant to cope with metal toxicity. For example, the production of growth hormones can increase the plant biomass and the root surface area or improve the uptake of essential nutrients like iron and phosphorus. Moreover, PGP traits confer the bacteria selective advantages for niche colonization against other competitors or pathogens (Rajkumar et al., 2012). Although several *Microbacterium* strains were positive in the ACC deaminase test, we could detect only genes coding for tryptophan synthase beta subunit. ACC deaminase is responsible for the degradation of ethylene, a plant hormone that is produced under various stress conditions including the presence of metals, other inorganic and organic chemicals (Arshad et al., 2007; Glick, 2014). ACC deaminase and tryptophan synthase belong to the same family, pyridoxal 5′-phosphate (PLP)-dependent enzymes. Even though ACC is the only supplied nitrogen source, traces of nitrogen might still support the growth of some strains and the results might be interpreted as false positives. Alternatively, the tested bacteria might have a PLP-dependent deaminase with non-specific ACC deaminase activity or use alternative ACC degradation pathways (Li et al., 2015). Even though, we could detect genes related to phosphorus metabolism in all genomes, not all *Microbacterium* strains were able to solubilize phosphate *in vitro*. The discrepancy between the results of the *in vitro* test and the *in silico* analysis might be due to the fact that phosphorus solubilization can be carried out using different strategies and pathways, which might not be activated under these specific conditions. The mechanisms leading to phosphorus mobilization influence also the mobility of other metals present in the soil. Bacteria can alter the absorption equilibria through the secretion of protons, organic anions, phosphatases and chelating molecules including siderophores (Richardson and Simpson, 2011). Some of them possess the PHO regulon, which controls the use of inorganic phosphate (Pi). This is a two-component regulatory system that is activated during Pi starvation and seems to be involved in the expression of genes related to metal stress as well (Baek and Lee, 2006; Santos-Beneit, 2015).

Heavy metal resistance tests showed that on average *Microbacterium* isolated from metal contaminated sites were more resistant to Zn, Pb, and Cd than the one isolated from “clean” sites. Moreover, the *Microbacterium* from contaminated sites possess more genes related to heavy metal homeostasis and resistance. The overabundance of genes conferring resistance to heavy metals and other stresses (e.g., nitrate, organic solvents, and pH) was also reported in a heavy metal contaminated groundwater microbial community (Hemme et al., 2010). Metallo-sensors function as co-repressors leading to the downregulation of genes for metal uptake and they stimulate the activation of genes related to metal efflux and storage. For instance, they control the activation of membrane transporters, channels and efflux pumps to reduce the overall uptake of potentially toxic metals. In addition, metallo-sensing regulators influence the production of secondary metabolites as siderophores and other chelators (Nies, 2003). The presence of resistance genes and metallo-sensing regulators seem to be essential factors for the adaptation to heavy metal contaminated environments (Hemme et al., 2016; Locatelli et al., 2016).

On the other hand, the ability to change the metal mobility in a contaminated soil seems to be widespread among all tested *Microbacterium* spp. The mobilization effect seems to be caused by a modification of the pH and by the production of specific compounds. Although the nature of such metabolites is still unclear, they could either be organic acids, specific ligands or siderophores with a broader metal specificity and able to chelate metals other than iron (Sessitsch et al., 2013; Johnstone and Nolan, 2015). Despite the promising results of the *in vitro* mobilization tests, the phytoremediation experiments did not provide the expected outcome. The inoculated microbes did not cause a significant change in the metal accumulation in *N. caerulescens* shoots. The weak mobilizer *M. azadirachtae* ARN176 induced an immobilization effect causing the decrease of some metals both in the shoots and roots of *B. napus*. On the other hand, the two best mobilizers, *M. hydrocarbonoxydans* SA35 and *Microbacterium* sp. 1.5R had limited or no influence on the accumulation of metals in *B. napus*.). The differences between the two plants might be due to the fact that *N. caerulescens* has a highly efficient regulation system that allows the plant to tolerate and accumulate very high metal concentrations (Halimaa et al., 2014; Mourato et al., 2015). Another important point to consider is the colonization efficiency of the inoculated *Microbacterium* strains. Since we used a non-sterile soil, these bacteria have to compete with the already existing microbial communities and find a way to successfully establish themselves in the new environment. *Microbacterium* strains were often isolated from plants grown in contaminated and non-contaminated soils (Behrendt et al., 2001; Kuffner et al., 2010). Nevertheless, the actual concentration and the metal-mobilizing effects of these bacteria was perhaps too small to provide excess amounts of metals readily available for plant uptake and translocation into leaf tissues. In this experimental set up, the concentration of the strains *in planta* as well as the mobilization of metals might be too small to be observed. Further experiments are required to assess the survival rate of these *Microbacterium* strains in the chosen contaminated soil and their ability to colonize *N. caerulescens* and *B. napus* plants.

The selection of bacterial isolates based on their genetic potential and their performance under laboratory conditions is unfortunately not always sufficient to predict their behavior under natural conditions. In fact, this process is influenced by several biotic (e.g., colonization ability and plant genotype) and abiotic factors (e.g., contamination levels) that should be considered. The investigation of the genomic repertoire and the chemical analysis of selected metabolites combined with microbial community studies may contribute to a better understanding of plant-microbe interactions and their adaptation mechanisms to harsh environments.

## Data Availability Statement

The datasets generated for this study can be found in the NCBI with accession number CP018151.

## Author Contributions

AS, GB, and EC designed the study. LA, EC, and GB contributed to the bioinformatics analysis. EC, CH, and MP performed the experiments. SW and KS isolated some of the strains used in this study. EC, GB, and LA wrote the manuscript. All authors read and approved the final manuscript.

## Conflict of Interest

The authors declare that the research was conducted in the absence of any commercial or financial relationships that could be construed as a potential conflict of interest.

## Footnotes

1 https://CRAN.R-project.org/package=vegan

2 https://rdrr.io/cran/mvabund/

3 http://mibig.secondarymetabolites.org/
