# Supplementary material for: Comparative Genomics of Microbacterium Species to Reveal Diversity, Potential for Secondary Metabolites and Heavy Metal Resistance
Source: Front Microbiol. 2020 Aug 4;11:1869. doi: 10.3389/fmicb.2020.01869 (PMC7438953; doi:10.3389/fmicb.2020.01869)
Supplement: Supplementary file 1 [file Data_Sheet_1.docx]

**Supplementary material to:**

Comparative genomics of *Microbacterium* species to reveal diversity, potential for secondary metabolites and heavy metal resistance

Erika Corretto^1⸹^, Livio Antonielli^1^, Angela Sessitsch^1^, Christoph Höfer^2⸸⸸^, Markus Puschenreiter^2^, Siegrid Widhalm^1^, Karivaradharajan Swarnalakshmi^3^, and Günter Brader^1^*

^1^Bioresouces Unit, Center for Health and Bioresources, AIT Austrian Institute of Technology GmbH, Tulln, Austria

^2^Institute of Soil Research, Department of Forest and Soil Sciences, University of Natural Resources and Life Sciences Vienna, Tulln, Austria

^3^Division of Microbiology, Indian Agricultural Research Institute, New Delhi, India

*** Correspondence:**Günter Brader
[Guenter.Brader@ait.ac.at](mailto:Guenter.Brader@ait.ac.at)

^⸹^ Present address: Faculty of Science and Technology, Free University of Bozen-Bolzano, Bolzano, Italy

^⸸⸸^ Present address: Institute of Biogeochemistry and Pollutant Dynamics, Department of Environmental Systems Science, ETH Zürich, Switzerland

# Tables and Figure legends

**Table S1.** General features of the 70 *Microbacterium* genomes used in this study. CDSs have been identified in Prokka (Seemann, 2014). Isolates in bold were cultivated and tested in this study. Strains isolated from heavy metal contaminated sites are marked with *. The other genomes available in the NCBI database belonging to strains isolated from contaminated site are marked with #.

| **Organism** | **Strain** | **Contigs** | **Size (Mb)** | **GC%** | **CDSs** | **rRNAs** | **BioProject** |
| --- | --- | --- | --- | --- | --- | --- | --- |
| ***M. azadirachtae*** | **ARN176*** | 40 | 4.24 | 70.1 | 3908 | 3 | PRJNA270387 |
| ***M. azadirachtae*** | **DSM 23848** | 86 | 4.04 | 70.5 | 3750 | 3 | PRJNA269724 |
| *M. chocolatum* | SIT 101 | 26 | 3.06 | 70.0 | 2914 | 5 | PRJNA280877 |
| *M. enclense* | NIO-1002 | 15 | 3.67 | 70.3 | 3355 | 6 | PRJNA302088 |
| ***M. foliorum*** | **DSM 12966** | 46 | 3.56 | 68.7 | 3324 | 5 | PRJNA269724 |
| ***M. ginsengisoli*** | **DSM 18659** | 80 | 3.05 | 70.2 | 2965 | 3 | PRJNA270387 |
| *M. gubbeenense* | DSM 15944 | 74 | 3.02 | 68.1 | 2800 | 9 | PRJNA185602 |
| *M. hominis* | LCDC 84-0209 | 73 | 3.51 | 70.9 | 3279 | 3 | PRJNA306491 |
| *M. hominis* | NBRC 15708 | 52 | 3.50 | 71.0 | 3209 | 3 | PRJDB1325 |
| ***M. hydrocarbonoxydans*** | **SA35*** | 10 | 3.95 | 68.5 | 3663 | 5 | PRJNA270387 |
| *M. indicum* | DSM 19969 | 28 | 2.81 | 71.4 | 2615 | 4 | PRJNA188869 |
| ***M. ketosireducens*** | **DSM 12510** | 57 | 3.92 | 70.3 | 3479 | 3 | PRJNA270387 |
| *M. laevaniformans* | LCDC 91-0039 | 59 | 3.00 | 69.4 | 2874 | 3 | PRJNA306496 |
| *M. (hominis) laevaniformans†* | TPW29 | 28 | 2.89 | 69.4 | 2717 | 3 | PRJNA269640 |
| *M. luticocti* | DSM 19459 | 27 | 3.12 | 70.7 | 2802 | 4 | PRJNA188870 |
| *M. mangrovi* | MUSC 115 | 55 | 4.42 | 70.0 | 4054 | 3 | PRJNA261100 |
| *M. oleivorans* | NBRC 103075 | 20 | 2.98 | 69.0 | 2852 | 3 | PRJDB453 |
| *M. oleivorans* | RIT293 | 11 | 2.90 | 69.0 | 2781 | 4 | PRJNA239285 |
| *M. (paraoxydans) oleivorans†* | 77MFTsu3.2 | 7 | 3.48 | 69.5 | 3502 | 5 | PRJNA185292 |
| ***M. oxydans*** | **BEL4b*** | 26 | 3.81 | 68.3 | 3589 | 5 | PRJNA270387 |
| ***M. oxydans*** | **BEL163*** | 30 | 3.69 | 68.0 | 3545 | 4 | PRJNA269724 |
| *M. paraoxydans* | DH1b | 47 | 3.62 | 70.2 | 3517 | 3 | PRJNA226198 |
| *M. profundi* | Shh49# | 12 | 3.37 | 66.5 | 3229 | 3 | PRJNA257114 |
| *M. testaceum* | StLB037 | 1 | 3.98 | 70.3 | 3633 | 6 | PRJDA62249 |
| ***M.trichothecenolyticum*** | **DSM 8608** | 41 | 4.52 | 70.2 | 4075 | 3 | PRJNA270387 |
| *M. yannicii* | PS01 | 67 | 3.95 | 69.5 | 3669 | 3 | PRJNA159261 |
| ***Microbacterium* sp.** | **1.5R** | 1 | 3.62 | 68.1 | 3433 | 6 | PRJNA352825 |
| *Microbacterium* sp. | 11MF | 3 | 2.91 | 69.9 | 2796 | 3 | PRJNA187941 |
| *Microbacterium* sp. | 292MF | 7 | 4.07 | 70.7 | 3748 | 4 | PRJNA183360 |
| *Microbacterium* sp. | 3J1 | 30 | 3.40 | 69.1 | 3268 | 4 | PRJEB8445 |
| *Microbacterium* sp. | Ag1 | 34 | 3.88 | 68.2 | 3790 | 3 | PRJNA281381 |
| *Microbacterium* sp. | CF335 | 4 | 3.02 | 70.8 | 2795 | 6 | PRJNA247513 |
| *Microbacterium* sp. | CGR1 | 1 | 3.63 | 68.0 | 3446 | 6 | PRJNA291433 |
| *Microbacterium* sp. | CH1 | 24 | 3.54 | 70.2 | 3429 | 3 | PRJNA305940 |
| *Microbacterium* sp. | CH12i | 21 | 3.35 | 63.8 | 3629 | 6 | PRJNA242700 |
| *Microbacterium* sp. | Cr-K1W# | 44 | 3.91 | 68.6 | 3731 | 12 | PRJNA236112 |
| *Microbacterium* sp. | Cr-K20# | 82 | 3.91 | 68.6 | 3731 | 12 | PRJNA236112 |
| *Microbacterium* sp. | Cr-K29# | 49 | 3.79 | 68.3 | 3582 | 12 | PRJNA236112 |
| *Microbacterium* sp. | Cr-K32# | 30 | 3.87 | 68.3 | 3633 | 12 | PRJNA236112 |
| *Microbacterium* sp. | G3 | 14 | 3.69 | 69.3 | 3490 | 13 | PRJEB7582 |
| *Microbacterium* sp. | GCS4 | 7 | 3.65 | 69.5 | 3490 | 3 | PRJNA291338 |
| *Microbacterium* sp. | Leaf151 | 7 | 3.61 | 69.8 | 3308 | 5 | PRJNA297956 |
| *Microbacterium* sp. | Leaf159 | 5 | 3.89 | 68.1 | 3693 | 3 | PRJNA297956 |
| *Microbacterium* sp. | Leaf161 | 6 | 3.63 | 67.9 | 3471 | 5 | PRJNA297956 |
| *Microbacterium* sp. | Leaf179 | 8 | 3.53 | 70.3 | 3264 | 3 | PRJNA297956 |
| *Microbacterium* sp. | Leaf203 | 36 | 3.45 | 69.9 | 3216 | 3 | PRJNA297956 |
| *Microbacterium* sp. | Leaf288 | 17 | 4.75 | 70.0 | 4139 | 3 | PRJNA297956 |
| *Microbacterium* sp. | Leaf320 | 9 | 3.87 | 67.9 | 3765 | 5 | PRJNA297956 |
| *Microbacterium* sp. | Leaf347 | 34 | 2.94 | 70.4 | 2807 | 3 | PRJNA297956 |
| *Microbacterium* sp. | Leaf351 | 38 | 2.89 | 70.2 | 2900 | 3 | PRJNA297956 |
| *Microbacterium* sp. | Leaf436 | 6 | 3.37 | 70.6 | 3139 | 3 | PRJNA297956 |
| *Microbacterium* sp. | MEJ108Y | 43 | 3.67 | 68.0 | 3533 | 3 | PRJNA270652 |
| *Microbacterium* sp. | No. 7 | 3 | 4.83^a^ | 71.0 | 4242 | 6 | PRJNA295341 |
| *Microbacterium* sp. | oral taxon 186 str.F0373 | 7 | 3.17 | 69.2 | 3003 | 6 | PRJNA169458 |
| *Microbacterium* sp. | PAMC28756/HSR44 | 1 | 3.54 | 70.4 | 3363 | 6 | PRJNA310789 |
| *Microbacterium* sp. | Root53 | 26 | 2.91 | 71.9 | 2729 | 3 | PRJNA297942 |
| *Microbacterium* sp. | Root61 | 2 | 4.02 | 68.7 | 3724 | 3 | PRJNA297942 |
| *Microbacterium* sp. | Root166 | 5 | 3.46 | 69.8 | 3158 | 3 | PRJNA297942 |
| *Microbacterium* sp. | Root180 | 5 | 3.35 | 70.0 | 3097 | 4 | PRJNA297942 |
| *Microbacterium* sp. | Root280D1 | 4 | 3.59 | 68.1 | 3468 | 5 | PRJNA297942 |
| *Microbacterium* sp. | Root322 | 3 | 3.60 | 68.5 | 3417 | 3 | PRJNA297942 |
| *Microbacterium* sp. | Root553 | 4 | 3.54 | 68.9 | 3331 | 5 | PRJNA297942 |
| *Microbacterium* sp. | Root1433D1 | 6 | 3.56 | 68.5 | 3391 | 3 | PRJNA297942 |
| ***Microbacterium* sp.** | **SA39*** | 39 | 3.86 | 68.3 | 3732 | 4 | PRJNA270387 |
| *Microbacterium* sp. | TS-1 | 3 | 3.40 | 70.2 | 3165 | 3 | PRJDB1140 |
| *Microbacterium* sp. | UCD-TDU | 8 | 3.75 | 68.4 | 3661 | 5 | PRJNA178255 |
| *Microbacterium* sp. | UNC423CL45Tsu | 17 | 3.11 | 70.9 | 2921 | 3 | PRJNA205976 |
| *Microbacterium* sp. | UNCCL10 | 15 | 3.11 | 70.9 | 2924 | 3 | PRJNA234875 |
| *Microbacterium* sp. | URHA0036 | 21 | 4.03 | 67.5 | 3818 | 3 | PRJNA190817 |
| *Microbacterium* sp. | XT11 | 1 | 3.48 | 69.4 | 3339 | 6 | PRJNA286063 |
| **Average** |  |  | 3.56 | 69.4 | 3369 |  |  |

**Table S2.** List of additional *Microbacterium* genomes used for the 16S rRNA gene tree of figure S1.

| **Assembly accession** | **Organism** |
| --- | --- |
| GCA_007988825.1 | *Microbacterium aerolatum* NBRC 103071 |
| GCA_002563955.1 | *Microbacterium agarici* DSM 21798 |
| GCA_011046975.1 | *Microbacterium amylolyticum* DSM 24221 |
| GCA_001662775.1 | *Microbacterium arborescens* ND21 |
| GCA_003339665.1 | *Microbacterium arborescens* RCB1 |
| GCA_001974985.1 | *Microbacterium aurum* KACC 15219 |
| GCA_003386675.1 | *Microbacterium chocolatum* 1320 |
| GCA_011761445.1 | *Microbacterium endophyticum* CECT 8354 |
| GCA_011047135.1 | *Microbacterium endophyticum* DSM 27099 |
| GCA_900163815.1 | *Microbacterium esteraromaticum* B Mb 05.01 |
| GCA_002024885.1 | *Microbacterium foliorum* 122 |
| GCA_006385575.1 | *Microbacterium foliorum* M2 |
| GCA_003367705.1 | *Microbacterium foliorum* NRRL B-24224 |
| GCA_003610275.1 | *Microbacterium ginsengiterrae* ZKA15 |
| GCA_011761265.1 | *Microbacterium halimionae* CECT 8593 |
| GCA_003569805.1 | *Microbacterium halotolerans* YIM 70130 |
| GCA_008017415.1 | *Microbacterium hatanonis* JCM14558 |
| GCA_002843965.1 | *Microbacterium hominis* SJTG1 |
| GCA_900105715.1 | *Microbacterium humi* DSM 21799 |
| GCA_011752045.1 | *Microbacterium hydrocarbonoxydans* C1-2 |
| GCA_900105205.1 | *Microbacterium hydrocarbonoxydans* DSM 16089 |
| GCA_004854025.1 | *Microbacterium hydrothermale* BPSAC84 |
| GCA_002872075.1 | *Microbacterium kitamiense* Sa12 |
| GCA_006783905.1 | *Microbacterium kyungheense* DSM 105492 |
| GCA_006716815.1 | *Microbacterium lacticum* DSM 20427 |
| GCA_002812805.1 | *Microbacterium lacus* SDZm4 |
| GCA_004794085.1 | *Microbacterium laevaniformans* NM46_B2-13 |
| GCA_003991875.1 | *Microbacterium lemovicicum* Viu22 |
| GCA_006788875.1 | *Microbacterium lindanitolerans* DSM 22422 |
| GCA_006539765.1 | *Microbacterium liquefaciens* NBRC 15037 |
| GCA_008868125.1 | *Microbacterium maritypicum* DSM 12512 |
| GCA_008017445.1 | *Microbacterium mitrae* M4-8 |
| GCA_001644225.1 | *Microbacterium oleivorans* CD11_3 |
| GCA_004358865.1 | *Microbacterium oleivorans* F-B2 |
| GCA_001691565.1 | *Microbacterium oleivorans* Wellendorf |
| GCA_001975955.2 | *Microbacterium oleivorans* A9 |
| GCA_009735645.1 | *Microbacterium oryzae* MB-10 |
| GCA_008868025.1 | *Microbacterium oxydans* DSM 20578 |
| GCA_012030385.1 | *Microbacterium oxydans* B19 |
| GCA_003991855.1 | *Microbacterium oxydans* HG3 |
| GCA_900095745.1 | *Microbacterium oxydans* Moxy_1 |
| GCA_006540085.1 | *Microbacterium oxydans* NBRC 15586 |
| GCA_004000565.1 | *Microbacterium oxydans* VIU2A |
| GCA_007679925.1 | *Microbacterium paludicola* DE0063 |
| GCA_007679895.1 | *Microbacterium paraoxydans* DE0066 |
| GCA_900105335.1 | *Microbacterium paraoxydans* DSM 15019 |
| GCA_004135285.1 | *Microbacterium protaetiae* DFW100M-13 |
| GCA_900100885.1 | *Microbacterium pygmaeum* DSM 23142 |
| GCA_008710705.1 | *Microbacterium radiodurans* DSM 25564 |
| GCA_008710745.1 | *Microbacterium rhizomatis* JCM 30598 |
| GCA_008017435.1 | *Microbacterium saccharophilum* K-1 |
| GCA_007992455.1 | *Microbacterium saccharophilum* NBRC 108778 |
| GCA_900113885.1 | *Microbacterium saccharophilum* UN80MFSha3.1 |
| GCA_006716345.1 | *Microbacterium saperdae* DSM 20169 |
| GCA_001689915.1 | *Microbacterium sediminis* YLB-01 |
| GCA_003327285.1 | *Microbacterium sorbitolivorans* .15228 |
| GCA_003931875.1 | *Microbacterium* sp. 10M-3 |
| GCA_003384875.1 | *Microbacterium* sp. 1294 |
| GCA_008271365.1 | *Microbacterium* sp. 1S1 |
| GCA_005502885.1 | *Microbacterium* sp. 2FI |
| GCA_004402135.1 | *Microbacterium* sp. 3H14 |
| GCA_005503465.1 | *Microbacterium* sp. 4NA327F11 |
| GCA_011046485.1 | *Microbacterium* sp. 4R-513 |
| GCA_900100525.1 | *Microbacterium* sp. 77mftsu3.1 |
| GCA_902506375.1 | *Microbacterium* sp. 8M |
| GCA_004794515.1 | *Microbacterium* sp. A20 |
| GCA_003850245.1 | *Microbacterium* sp. ABRD_28 |
| GCA_003635115.1 | *Microbacterium* sp. AG1240 |
| GCA_003385695.1 | *Microbacterium* sp. AG157 |
| GCA_003610405.1 | *Microbacterium* sp. AG238 |
| GCA_003634765.1 | *Microbacterium* sp. AG790 |
| GCA_008369345.1 | *Microbacterium* sp. ANT_H45B |
| GCA_010634855.1 | *Microbacterium* sp. B35-30 |
| GCA_011754135.1 | *Microbacterium* sp. Be9 |
| GCA_005502545.1 | *Microbacterium* sp. Beta |
| GCA_007679045.1 | *Microbacterium* sp. BH-3-3-3 DE0130 |
| GCA_007673975.1 | *Microbacterium* sp. BH-3-3-3 DE0319 |
| GCA_001792815.1 | *Microbacterium* sp. BH-3-3-3 |
| GCA_004216995.1 | *Microbacterium* sp. BK664 |
| GCA_004362195.1 | *Microbacterium* sp. BK668 |
| GCA_002812725.1 | *Microbacterium* sp. BR1 |
| GCA_007954505.1 | *Microbacterium* sp. CBA3102 |
| GCA_011326725.1 | *Microbacterium* sp. CBS5P-1 |
| GCA_900114345.1 | *Microbacterium* sp. cf046 |
| GCA_900102175.1 | *Microbacterium* sp. cf332 |
| GCA_012524205.1 | *Microbacterium* sp. CFH 90308 |
| GCA_003626735.1 | *Microbacterium* sp. CGR2 |
| GCA_002993305.1 | *Microbacterium* sp. str. 'China' |
| GCA_002911615.1 | *Microbacterium* sp. CJ77 |
| GCA_900114965.1 | *Microbacterium* sp. cl127 |
| GCA_900108235.1 | *Microbacterium* sp. Cl12a |
| GCA_900103075.1 | *Microbacterium* sp. cl140 |
| GCA_900107955.1 | *Microbacterium* sp. Cl152 |
| GCA_001984105.1 | *Microbacterium* sp. CSI-V |
| GCA_004570445.1 | *Microbacterium* sp. dk485 |
| GCA_008868005.1 | *Microbacterium* sp. G1 |
| GCA_003095395.1 | *Microbacterium* sp. Gd 4-13 |
| GCA_001650405.1 | *Microbacterium* sp. H83 |
| GCA_001639925.1 | *Microbacterium* sp. HM58-2 |
| GCA_011751785.1 | *Microbacterium* sp. HY54 |
| GCA_011751765.1 | *Microbacterium* sp. HY60 |
| GCA_011751745.1 | *Microbacterium* sp. HY82 |
| GCA_011751795.1 | *Microbacterium* sp. HY89 |
| GCA_009360775.1 | *Microbacterium* sp. isolate GV_Bin_6 |
| GCA_003248655.1 | *Microbacterium* sp. isolate S2_005_003_R2_44 |
| GCA_003335195.1 | *Microbacterium* sp. JB110 |
| GCA_004794535.1 | *Microbacterium* sp. K19 |
| GCA_004794525.1 | *Microbacterium* sp. K21 |
| GCA_004794435.1 | *Microbacterium* sp. K27 |
| GCA_004794385.1 | *Microbacterium* sp. K5D |
| GCA_003121305.1 | *Microbacterium* sp. KCTC 39802 |
| GCA_008727775.1 | *Microbacterium* sp. L-031 |
| GCA_008725615.1 | *Microbacterium* sp. L-033 |
| GCA_003999995.1 | *Microbacterium* sp. LAM7116 |
| GCA_001866135.1 | *Microbacterium* sp. LCT-H2 |
| GCA_002114135.1 | *Microbacterium* sp. LEMMJ01 |
| GCA_900102005.1 | *Microbacterium* sp. LKL04 |
| GCA_009758255.1 | *Microbacterium* sp. MAH-37 |
| GCA_900292075.1 | *Microbacterium* sp. Marseille-P5731 |
| GCA_012847295.1 | *Microbacterium* sp. MF43 |
| GCA_002979795.1 | *Microbacterium* sp. MYb24 |
| GCA_002979695.1 | *Microbacterium* sp. MYb32 |
| GCA_002975375.1 | *Microbacterium* sp. MYb40 |
| GCA_002975345.1 | *Microbacterium* sp. MYb43 |
| GCA_002979655.1 | *Microbacterium* sp. MYb45 |
| GCA_002979595.1 | *Microbacterium* sp. MYb50 |
| GCA_002979515.1 | *Microbacterium* sp. MYb54 |
| GCA_002979475.1 | *Microbacterium* sp. MYb62 |
| GCA_002979495.1 | *Microbacterium* sp. MYb64 |
| GCA_002979435.1 | *Microbacterium* sp. MYb66 |
| GCA_002979415.1 | *Microbacterium* sp. MYb72 |
| GCA_004366135.1 | *Microbacterium* sp. NFIX05 |
| GCA_005502335.1 | *Microbacterium* sp. O |
| GCA_004337555.1 | *Microbacterium* sp. PI-1 |
| GCA_003293595.1 | *Microbacterium* sp. PM5 |
| GCA_900156435.1 | *Microbacterium* sp. RU1D |
| GCA_900155915.1 | *Microbacterium* sp. RU33B |
| GCA_900104435.1 | *Microbacterium* sp. ru370.1 |
| GCA_002920555.1 | *Microbacterium* sp. Ru50 |
| GCA_900156455.1 | *Microbacterium* sp. RURRCA19A |
| GCA_009858275.1 | *Microbacterium* sp. SAI-030 |
| GCA_005491085.2 | *Microbacterium* sp. SGAir0570 |
| GCA_006715145.1 | *Microbacterium* sp. SLBN-146 |
| GCA_006715565.1 | *Microbacterium* sp. SLBN-154 |
| GCA_006715675.1 | *Microbacterium* sp. SLBN-158 |
| GCA_003289625.1 | *Microbacterium* sp. SMR1 |
| GCA_008727755.1 | *Microbacterium* sp. ST-M6 |
| GCA_009649635.1 | *Microbacterium* sp. SYP-A9085 |
| GCA_002362255.1 | *Microbacterium* sp. SZ1 Y1 |
| GCA_001619615.1 | *Microbacterium* sp. T32 |
| GCA_009832945.1 | *Microbacterium* sp. TL13 |
| GCA_001620065.1 | *Microbacterium* sp. TNHR37B |
| GCA_003075395.1 | *Microbacterium* sp. TPD7012 |
| GCA_002356155.1 | *Microbacterium* sp. TPU 359 |
| GCA_002871415.1 | *Microbacterium* sp. UMB0228 |
| GCA_003856715.1 | *Microbacterium* sp. Y-01 |
| GCA_002245215.1 | *Microbacterium* sp. Yaish 1 |
| GCA_002970975.1 | *Microbacterium* sp. YJYP303 |
| GCA_003254645.1 | *Microbacterium* sp. YZYP306 |
| GCA_002970955.1 | *Microbacterium* sp. YZYP518 |
| GCA_003651225.1 | *Microbacterium telephonicum* S2T63 |
| GCA_007679235.1 | *Microbacterium testaceum* DE0116 |
| GCA_007671785.1 | *Microbacterium testaceum* DE0590 |
| GCA_002899925.1 | *Microbacterium testaceum* KU313 |
| GCA_006539145.1 | *Microbacterium testaceum* NBRC 12675 |
| GCA_003386735.1 | *Microbacterium trichothecenolyticum* ZKA46 |
| GCA_011759705.1 | *Microbacterium ulmi* CECT 5976 |
| GCA_008017485.1 | *Microbacterium wangchenii* dk508 |
| GCA_004564355.1 | *Microbacterium wangchenii* dk512 |

**Table S3.** List of the 38 marker genes identified by Phylosift (Darling et al., 2014).

| **Gene** | **Description** |
| --- | --- |
| 16s_reps_bac | 16S rRNA gene |
| DNGNGWU00001 | ribosomal protein S2 rpsB |
| DNGNGWU00002 | ribosomal protein S10 rpsJ |
| DNGNGWU00003 | ribosomal protein L1 rplA |
| DNGNGWU00005 | translation initiation factor IF-2 |
| DNGNGWU00006 | metalloendopeptidase |
| DNGNGWU00007 | ribosomal protein L22 |
| DNGNGWU00009 | ribosomal protein L4/L1e rplD |
| DNGNGWU00010 | ribosomal protein L2 rplB |
| DNGNGWU00011 | ribosomal protein S9 rpsI |
| DNGNGWU00012 | ribosomal protein L3 rplC |
| DNGNGWU00013 | phenylalanyl-tRNA synthetase beta subunit |
| DNGNGWU00014 | ribosomal protein L14b/L23e rplN |
| DNGNGWU00015 | ribosomal protein S5 |
| DNGNGWU00016 | ribosomal protein S19 rpsS |
| DNGNGWU00017 | ribosomal protein S7 |
| DNGNGWU00018 | ribosomal protein L16/L10E rplP |
| DNGNGWU00019 | ribosomal protein S13 rpsM |
| DNGNGWU00020 | phenylalanyl-tRNA synthetase alpha subunit |
| DNGNGWU00021 | ribosomal protein L15 |
| DNGNGWU00022 | ribosomal protein L25/L23 |
| DNGNGWU00023 | ribosomal protein L6 rplF |
| DNGNGWU00024 | ribosomal protein L11 rplK |
| DNGNGWU00025 | ribosomal protein L5 rplE |
| DNGNGWU00026 | ribosomal protein S12/S23 |
| DNGNGWU00027 | ribosomal protein L29 |
| DNGNGWU00028 | ribosomal protein S3 rpsC |
| DNGNGWU00029 | ribosomal protein S11 rpsK |
| DNGNGWU00030 | ribosomal protein L10 |
| DNGNGWU00031 | ribosomal protein S8 |
| DNGNGWU00032 | tRNA pseudouridine synthase B |
| DNGNGWU00033 | ribosomal protein L18P/L5E |
| DNGNGWU00034 | ribosomal protein S15P/S13e |
| DNGNGWU00035 | Porphobilinogen deaminase |
| DNGNGWU00036 | ribosomal protein S17 |
| DNGNGWU00037 | ribosomal protein L13 rplM |
| DNGNGWU00039 | ribonuclease HII |
| DNGNGWU00040 | ribosomal protein L24 |

**Table S4.** Secondary metabolite gene clusters of the 70 *Microbacterium* genomes identified in antiSMASH 5.0 (Blin et al., 2019). Secondary metabolite gene clusters: Ter, terpenoids; PKS, type III polyketide synthase; NRPS, non-ribosomal peptide synthetase; RiPP, ribosomally synthesized post-translationally modified peptides; Sid, siderophore; Ect, ectoine; But, butyrolactone; Bac, bacteriocin; Res, resorcinol. Based on direct observation (ARN176) and literature data (DSM 19459, XT11), bacteria lacking the terpenoid cluster have white colonies (-/white); no information on the colony coloration could be retrieved for RIT293. The NRPS column includes both the “nrps” and “other” categories. The RiPP column includes lantipeptides, lassopeptides, ladderanes, thiopeptides and linaridin. Isolates in bold were tested in this study. Strains isolated from heavy metal contaminated sites are marked with *. The other genomes available in the NCBI database belonging to strains isolated from contaminated site are marked with #.

| **Strain** | **Ter** | **PKS** | **NRPS** | **RiPP** | **Sid** | **Ect** | **But** | **Bac** | **Res** | **Tot** |
| --- | --- | --- | --- | --- | --- | --- | --- | --- | --- | --- |
| **ARN176*** | -/white | 1 | 1 | - | - | - | - | - | - | 2 |
| **DSM 23848** | 1 | 1 | 2 | 1 | - | - | - | 1 | - | 6 |
| SIT 101 | 1 | 1 | - | - | - | - | - | - | - | 2 |
| NIO-1002 | 1 | 1 | - | 1 | - | - | - | - | - | 3 |
| **DSM 12966** | 1 | 1 | 1 | - | - | - | - | - | - | 3 |
| **DSM 18659** | 1 | - | - | - | - | - | - | - | - | 1 |
| DSM 15944 | 1 | - | 2 | - | - | 1 | - | - | - | 4 |
| LCDC 84-0209 | 1 | - | 1 | - | - | - | - | - | - | 2 |
| NBRC 15708 | 1 | - | 1 | - | - | - | - | - | - | 2 |
| **SA35*** | 1 | 1 | 1 | - | 1 | - | - | 1 | - | 5 |
| DSM 19969 | 1 | - | 1 | - | - | - | - | - | - | 2 |
| **DSM 12510** | 2 | 1 | 1 | - | 1 | - | - | - | 1 | 7 |
| LCDC 91-0039 | 1 | - | 2 | - | - | - | - | - | - | 3 |
| TPW29 | 1 | - | 1 | - | - | - | - | - | - | 2 |
| DSM 19459 | -/white | - | - | - | - | - | - | 1 | - | 1 |
| MUSC 115 | 1 | 1 | 1 | - | - | - | - | - | - | 3 |
| NBRC 103075 | 1 | 1 | 1 | - | - | - | - | - | - | 3 |
| RIT293 | 1 | 1 | 1 | - | - | - | - | - | - | 3 |
| 77MFTsu3.2 | 1 | 1 | - | - | - | - | - | - | - | 2 |
| **BEL4b*** | 1 | 1 | 1 | - | - | - | - | - | - | 3 |
| **BEL163*** | 1 | - | - | - | - | - | - | - | - | 1 |
| DH1b | 1 | 1 | - | - | - | - | - | - | - | 2 |
| Shh49# | 1 | 1 | 2 | - | - | 1 | - | - | - | 5 |
| StLB037 | 1 | 1 | 1 | - | - | - | - | - | - | 3 |
| **DSM 8608** | 1 | 1 | 2 | - | - | - | - | - | 1 | 5 |
| PS01 | 1 | 1 | 1 | - | - | - | - | - | - | 3 |
| **1.5R** | 1 | 1 | 1 | - | - | - | - | - | - | 3 |
| 11MF | 1 | 1 | - | - | - | - | - | - | - | 2 |
| 292MF | 1 | 1 | - | 1 | - | - | - | - | - | 3 |
| 3J1 | 1 | 1 | 1 | - | - | - | - | - | - | 3 |
| Ag1 | 1 | 1 | - | - | - | - | - | - | - | 2 |
| CF335 | 1 | 1 | 1 | - | - | - | - | - | - | 3 |
| CGR1 | 1 | 1 | 1 | - | - | - | - | - | - | 3 |
| CH1 | 1 | 1 | - | - | - | - | - | - | - | 2 |
| CH12i | 1 | 1 | - | - | - | - | - | - | - | 2 |
| Cr-K1W# | 1 | 1 | - | - | - | - | - | - | - | 2 |
| Cr-K20# | 1 | 1 | - | - | - | - | - | - | - | 2 |
| Cr-K29# | 1 | 1 | - | - | 1 | - | - | - | - | 3 |
| Cr-K32# | 1 | 1 | - | - | 1 | - | - | - | - | 3 |
| G3 | 1 | - | - | - | - | - | - | - | - | 1 |
| GCS4 | 1 | 1 | - | - | - | - | - | 1 | - | 3 |
| Leaf151 | 1 | 1 | 1 | - | - | - | - | - | - | 3 |
| Leaf159 | 1 | 1 | - | - | - | - | - | - | - | 2 |
| Leaf161 | 1 | 1 | 1 | 1 | - | - | - | - | - | 4 |
| Leaf179 | 1 | 1 | 1 | - | - | - | - | - | - | 3 |
| Leaf203 | 1 | 1 | 1 | - | - | - | - | - | - | 3 |
| Leaf288 | 1 | 1 | 2 | - | 1 | - | - | - | 1 | 6 |
| Leaf320 | 1 | 1 | 1 | - | - | - | - | - | - | 3 |
| Leaf347 | 1 | - | - | - | - | - | - | - | - | 1 |
| Leaf351 | 1 | - | - | - | - | - | - | - | - | 1 |
| Leaf436 | 1 | 1 | 1 | 1 | - | - | - | - | - | 4 |
| MEJ108Y | 1 | 1 | 1 | - | - | - | - | - | - | 3 |
| No. 7 | 1 | - | 3 | - | - | - | - | 1 | 1 | 5 |
| oral taxon 186 str.F0373 | 1 | - | 2 | - | - | - | - | - | - | 3 |
| PAMC28756/HSR44 | 1 | 1 | - | - | - | - | - | - | - | 2 |
| Root53 | - | - | - | - | - | - | - | - | - | 0 |
| Root61 | 1 | 1 | - | - | - | - | - | - | 1 | 3 |
| Root166 | 1 | 1 | - | - | 1 | - | - | - | 1 | 4 |
| Root180 | 1 | 1 | - | - | - | - | - | - | 1 | 3 |
| Root280D1 | 1 | 1 | 1 | - | - | - | - | - | - | 3 |
| Root322 | 1 | 1 | - | - | - | - | - | - | - | 2 |
| Root553 | 1 | 1 | 1 | - | - | - | - | - | - | 3 |
| Root1433D1 | 1 | 1 | - | - | - | - | 1 | - | - | 3 |
| SA39 | 1 | 1 | 1 | - | - | - | - | - | - | 3 |
| TS-1 | 1 | 1 | - | 1 | - | - | - | - | - | 3 |
| UCD-TDU | 1 | 1 | - | - | - | - | - | - | - | 2 |
| UNC423CL45Tsu | 1 | 1 | - | - | - | - | - | - | - | 2 |
| UNCCL10 | 1 | 1 | - | - | - | - | - | - | - | 2 |
| URHA0036 | 1 | 1 | - | - | - | - | - | - | - | 2 |
| XT11 | -/white | 1 | - | - | - | - | - | - | - | 1 |
|  | **Ter** | **PKS** | **NRPS** | **RiPP** | **Sid** | **Ect** | **But** | **Bac** | **Res** | **Tot** |
| **Genomes (num)** | 67 | 55 | 45 | 6 | 6 | 2 | 1 | 5 | 7 |  |
| **Genomes (%)** | 96 | 79 | 64 | 9 | 9 | 3 | 1 | 7 | 10 |  |

**Table S5.** Secondary metabolite gene clusters detected in antiSMASH 5.0 (Blin et al., 2019) within the siderophore category. Cluster 1: *M. hydrocarbonoxydans* SA35*, *M. ketosireducens* DSM 12510, *Microbacterium* sp. Leaf288 and sp. Root166; Cluster 2: *Microbacterium* sp. Cr-K29# and Cr-K32#. Bacteria isolated from heavy metal contaminated sites are marked with *. The other genomes available in the NCBI database belonging to strains isolated from contaminated site are marked with #.

| **Siderophore cluster description:** | Desferrioxamine_B_biosynthetic_gene_cluster (60-80% of genes show similarity) - MIBiG BGC0000941_c1 | |
| --- | --- | --- |
|  | **Genes** |  |
| *Cluster 1* | desA | L-lysine decarboxylase |
|  | desB | 1,5-diaminopentane monoxygenase |
|  | desC | acyl-CoA acyl transferase |
|  | desD | type C siderophore synthetase (homologous to IucC of *E. coli*) |
|  | acyII | penicillin acyclase 2 precursor |
|  | desC | acyl-CoA acyl transferase |
| *Cluster 2* | desA | L-lysine decarboxylase |
|  | desB | 1,5-diaminopentane monoxygenase |
|  | desC | acyl-CoA acyl transferase |
|  | desD | type C siderophore synthetase (homologous to IucC of *E. coli*) |

The two transporter genes (*desEF*), usually present in clusters for the production of desferrioxamine E in *Streptomyces* genomes, could not be identified in the *Microbacterium* genomes. In fact, among their ferric-siderophore transporters, none showed significant similarity to *desE* and only few showed low amino acid identity (30-40%) with *desF* and *cdtB*. These transporters were situated far from the *desABCD* cluster, with the exception of the closely related *Microbacterium* sp. Cr-K29 and Cr-K32, that have a ferric-siderophore transporter about 7 kb up-/down-stream, respectively.

**Table S6.** Secondary metabolite gene clusters detected in antiSMASH 5.0 (Blin et al., 2019) within the “nrps” category. NRPS domains: A, adenylation domain; ACPS, acyl carrier protein synthase domain; C, condensation domain; KR, keto-reductase domain; PCP, peptidyl carrier protein domain; PP, phosphopantetheine binding domain; TE, thioesterase domain. Predicted substrates of A domains: bht, beta-hydroxy-tyrosine; dhb, 2,3-dihydroxy-benzoic acid; dhpg, 3,5-dihydroxy-phenyl-glycin; hpg, 4-hydoxy-phenylglycine; phe, phenylalanine; ser, serine; thr, threonine; trp, tryptophan; tyr, tyrosine.

| **Organism** | **NRPS cluster description - MIBiG** | **Genes** | **NRPS domains** | **Predicted substrate of A domain** |
| --- | --- | --- | --- | --- |
| *M. gubbeenense* DSM 15944 | Griseobactin_biosynthetic_gene_cluster (29% of genes show similarity) - BGC0000368_c1 | | | |
|  |  | 4'-phosphopantetheinyl transferase Npt | ACPS | - |
|  |  | isochorismatase | - | - |
|  |  | 2,3-dihydro-2,3-dihydroxybenzoate dehydrogenase | KR | - |
|  |  | isochorismatase synthase DhbC | - | - |
|  |  | thioesterase superfamily protein | - | - |
|  |  | acetyl-CoA synthetase | A | - |
|  |  | phenyloxazoline synthase MbtB | C-A | hydrophobic-aliphatic |
|  |  | dimodular nrps | C-PCP-C-A-PCP | phe, trp, hpg, tyr, bht |
|  |  | linear gramicidin synthase subunit D | C-A-PCP | ser, thr, dhpg, hpg |
|  |  | phenyloxazoline synthase MbtB | C-A-PCP | hydrophilic |
|  |  | isochorismatase | PCP | - |
|  |  | 2,3-dihydroxybenzoate-AMP-ligase | A | dhb |
| *Microbacterium* sp. No.7 | Enterobactin_biosynthetic_gene_cluster (8% of genes show similarity) - BGC0000343_c1 | | | |
|  |  | 2,3-dihydro-2,3-dihydroxybenzoate dehydrogenase entA | KR | - |
|  |  | isochorismatase synthase DhbC (entC) | - | - |
|  |  | 2,3-dihydroxybenzoate-AMP-ligase (entE) | A | dhb |
|  |  | isochorismatase vibrobactin-specific | - | - |
|  |  | enterobactin synthase component F (entF) | C-A-PCP-TE | ser |
|  |  | hypothetical protein - phosphopantetheinyl transferase component of enterobactin synthesis | ACPS | - |
|  |  | acetoacetyl-CoA synthase NphT7 | - | - |
| *M. azadirachtae* DSM 23848 | - | acyl carrier protein | PCP | - |
|  |  | surfactin synthase subunit 1 | A | hydrophobic-aliphatic |
|  |  | peptide synthase | C | - |
|  |  | enterobactin/ferric enterobactin esterase | - | - |
| *M. yannicii* PS01 | - | holo-(acyl carrier protein) synthase 2 | ACPS | - |
|  |  | acyl carrier protein | PP | - |
|  |  | long-chain-fatty-acid AMP ligase (FadD29) | A | - |

**Table S7.** Genes for the synthesis of tryptophan (IAA main precursor) and for its conversion to IAA through the tryptamine pathway identified in RAST (Overbeek et al., 2014). The results of the test for the production of IAA are reported in the last row. Organisms: 1, *M. azadirachtae* ARN176; 2, *M. hydrocarbonoxydans* SA35; 3, *M. oxydans* BEL4b; 4, *M. oxydans* BEL163; 5, *Microbacterium* sp. SA39; 6, *M. azadirachtae* DSM 23848; 7, *M. foliorum* DSM 12966; 8, *M. ginsengisoli* DSM 18659; 9, *M. ketosireducens* DSM 12510; 10, *M. trichothecenolyticum* DSM 8608; 11, *Microbacterium* sp. 1.5R. +HM, bacteria isolated from heavy metal contaminated sites; -HM, bacteria isolated from non-contaminated sites.

| **Genes/Organisms** | **+HM** | | | | | **-HM** | | | | | |
| --- | --- | --- | --- | --- | --- | --- | --- | --- | --- | --- | --- |
|  | **1** | **2** | **3** | **4** | **5** | **6** | **7** | **8** | **9** | **10** | **11** |
| ***Tryptophan biosynthesis:*** |  |  |  |  |  |  |  |  |  |  |  |
| Anthranilate phosphoribosyltransferase (EC 2.4.2.18) | 1 | 1 | 1 | - | 1 | 1 | 1 | 1 | 1 | 1 | 1 |
| Tryptophan synthase alpha chain (EC 4.2.1.20) | 1 | 1 | 1 | 1 | 1 | 1 | 1 | 1 | 1 | 1 | 1 |
| Tryptophan synthase beta chain (EC 4.2.1.20) | 1 | 1 | 1 | 1 | 1 | 1 | 1 | 1 | 1 | 1 | 1 |
| ***Tryptamine pathway for IAA biosynthesis:*** |  |  |  |  |  |  |  |  |  |  |  |
| Aromatic-L-amino-acid decarboxylase (EC 4.1.1.28) | 1 | 1 | 2 | 1 | 2 | 1 | 2 | 1 | 2 | 1 | 2 |
| Monoamine oxidase (1.4.3.4) | 2 | 2 | 2 | 2 | 1 | 2 | 1 | 1 | 1 | 3 | 2 |
| **Test for IAA production** | + | + | - | + | + | + | - | + | - | + | - |

**Table S8.** Genes related to phosphorus mobilization and metabolism identified in RAST (Overbeek et al., 2014). The results of the test for phosphorus solubilization are reported in the last row. Organisms: 1, *M. azadirachtae* ARN176; 2, *M. hydrocarbonoxydans* SA35; 3, *M. oxydans* BEL4b; 4, *M. oxydans* BEL163; 5, *Microbacterium* sp. SA39; 6, *M. azadirachtae* DSM 23848; 7, *M. foliorum* DSM 12966; 8, *M. ginsengisoli* DSM 18659; 9, *M. ketosireducens* DSM 12510; 10, *M. trichothecenolyticum* DSM 8608; 11, *Microbacterium* sp. 1.5R. +HM, bacteria isolated from heavy metal contaminated sites; -HM, bacteria isolated from non-contaminated sites.

| **Genes/Organisms** | **+HM** | | | | | **-HM** | | | | | |
| --- | --- | --- | --- | --- | --- | --- | --- | --- | --- | --- | --- |
| ***RAST subsystem*** | **1** | **2** | **3** | **4** | **5** | **6** | **7** | **8** | **9** | **10** | **11** |
| *High affinity phosphate transporter and control of PHO regulon, PhoR-PhoB two-component regulatory system, Phosphate metabolism* |  |  |  |  |  |  |  |  |  |  |  |
| Phosphate ABC transporter, periplasmic phosphate-binding protein PstS (TC 3.A.1.7.1) | 1 | 1 | 2 | 1 | 1 | 2 | 1 | 2 | 1 | 1 | 1 |
| Phosphate regulon sensor protein PhoR (SphS) (EC 2.7.13.3) | 2 | 1 | 1 | 2 | 1 | 2 | 1 | 1 | 1 | 1 | 1 |
| Phosphate regulon transcriptional regulatory protein PhoB (SphR) | 1 | 1 | 1 | 2 | 3 | 2 | 1 | 2 | 1 | 2 | 2 |
| *High affinity phosphate transporter and control of PHO regulon, Phosphate metabolism* |  |  |  |  |  |  |  |  |  |  |  |
| Phosphate transport ATP-binding protein PstB (TC 3.A.1.7.1) | 1 | 1 | 2 | 1 | 1 | 1 | 1 | 2 | 1 | 1 | 1 |
| Phosphate transport system permease protein PstA (TC 3.A.1.7.1) | 1 | 1 | 2 | 1 | 1 | 1 | 1 | 2 | 1 | 1 | 1 |
| Phosphate transport system permease protein PstC (TC 3.A.1.7.1) | 1 | 1 | 2 | 1 | 1 | 1 | 1 | 1 | 1 | 1 | 1 |
| Phosphate transport system regulatory protein PhoU | 1 | 1 | 1 | 1 | 1 | 1 | 1 | 1 | 1 | 1 | 1 |
| *High affinity phosphate transporter and control of PHO regulon, Phosphate metabolism, Polyphosphate, Purine conversions* |  |  |  |  |  |  |  |  |  |  |  |
| Polyphosphate kinase (EC 2.7.4.1) | 1 | 1 | 1 | 2 | 1 | 1 | 1 | 1 | 1 | 1 | 1 |
| *Polyphosphate* |  |  |  |  |  |  |  |  |  |  |  |
| Polyphosphate kinase 2 (EC 2.7.4.1) | - | - | - | 1 | - | - | - | - | - | 1 | - |
| *Phosphate metabolism* |  |  |  |  |  |  |  |  |  |  |  |
| Alkaline phosphatase (EC 3.1.3.1) | 1 | - | - | 1 | 1 | - | - | - | 2 | 2 | - |
| Inorganic pyrophosphatase (EC 3.6.1.1) | 1 | 1 | 1 | 1 | 1 | 1 | 1 | 1 | 1 | 1 | 1 |
| NAD(P) transhydrogenase alpha subunit (EC 1.6.1.2) | 1 | - | - | - | 1 | 1 | - | - | 1 | 2 | - |
| NAD(P) transhydrogenase subunit beta (EC 1.6.1.2) | 1 | - | - | - | 1 | 1 | - | - | 1 | 2 | - |
| Predicted ATPase related to phosphate starvation-inducible protein PhoH | 2 | 2 | 2 | 2 | 2 | 2 | 2 | 3 | 2 | 2 | 2 |
| Probable low-affinity inorganic phosphate transporter | 2 | 2 | 1 | 1 | 1 | 2 | 1 | - | 1 | 1 | 1 |
| *Phosphate metabolism, Polyphosphate* |  |  |  |  |  |  |  |  |  |  |  |
| Exopolyphosphatase (EC 3.6.1.11) | 1 | 1 | 1 | 1 | 1 | 1 | 1 | 1 | 1 | 1 | 1 |
| *Entner-Doudoroff Pathway, Glycolysis and Gluconeogenesis, Polyphosphate* |  |  |  |  |  |  |  |  |  |  |  |
| Polyphosphate glucokinase (EC 2.7.1.63) | 1 | 1 | 1 | 1 | 1 | 1 | 1 | 1 | 1 | 1 | 1 |
| **Test for P solubilization** | + | + | - | + | - | + | + | + | - | + | - |

**Table S9.** Genes related to iron acquisition and metabolism identified in RAST (Overbeek et al., 2014). The results of the tests for siderophore production and iron mobilization are reported in the last row. Organisms: 1, *M. azadirachtae* ARN176; 2, *M. hydrocarbonoxydans* SA35; 3, *M. oxydans* BEL4b; 4, *M. oxydans* BEL163; 5, *Microbacterium* sp. SA39; 6, *M. azadirachtae* DSM 23848; 7, *M. foliorum* DSM 12966; 8, *M. ginsengisoli* DSM 18659; 9, *M. ketosireducens* DSM 12510; 10, *M. trichothecenolyticum* DSM 8608; 11, *Microbacterium* sp. 1.5R. (*), this gene is coding for siderophore interacting protein. even though is included in desferrioxamine biosynthesis category. +HM, bacteria isolated from heavy metal contaminated sites; -HM, bacteria isolated from non-contaminated sites.

| **Genes/Organisms** | **+HM** | | | | | **-HM** | | | | | |
| --- | --- | --- | --- | --- | --- | --- | --- | --- | --- | --- | --- |
| ***RAST subsystem*** | **1** | **2** | **3** | **4** | **5** | **6** | **7** | **8** | **9** | **10** | **11** |
| Petrobactin-mediated iron uptake system | 7 | - | - | 5 | 5 | 5 | - | - | 1 | - | 5 |
| Siderophore Aerobactin (only transporters) | 4 | - | - | - | - | - | - | - | - | - | - |
| Iron acquisition in Streptococcus | 3 | - | - | - | - | 3 | - | 3 | - | 3 | - |
| Ferrous iron transporter EfeUOB, low-pH-induced | 3 | 3 | 3 | 3 | 3 | 3 | 3 | 3 | - | 3 | 3 |
| Siderophore transporters | 3 | 3 | 16 | 5 | 9 | 7 | 18 | 6 | 4 | 3 | 10 |
| desferrioxamine biosynthesis | - | 4 | - | - | - | - | - | 1* | 4 | - | - |
| Putative Siderophore biosynthesis | 1 | - | - | 1 | - | - | - | - | - | - | - |
| Putative Siderophore interacting protein | 1 | 1 | 1 | 1 | 1 | 1 | 5 | - | 3 | 3 | 4 |
| **Total** | 22 | 11 | 20 | 15 | 18 | 19 | 26 | 13 | 12 | 12 | 22 |
| **Test for siderophore production** | - | - | + | - | - | - | + | + | + | - | + |
| **Mobilized iron (mg kg-1 soil)** | 10.1 | 23.5 | 5.6 | 9.9 | 9.7 | 8.8 | 8.6 | 9.0 | 14.4 | 18.0 | 19.6 |

**Table S10.** Genes related to metal homeostasis (HM genes) and metalloregulators. Organisms: 1, *M. azadirachtae* ARN176; 2, *M. hydrocarbonoxydans* SA35; 3, *M. oxydans* BEL4b; 4, *M. oxydans* BEL163; 5, *Microbacterium* sp. SA39; 6, *M. azadirachtae* DSM 23848; 7, *M. foliorum* DSM 12966; 8, *M. ginsengisoli* DSM 18659; 9, *M. ketosireducens* DSM 12510; 10, *M. trichothecenolyticum* DSM 8608; 11, *Microbacterium* sp. 1.5R. +HM, bacteria isolated from heavy metal contaminated sites; -HM, bacteria isolated from non-contaminated sites.

|  | **Genes/Organisms** | **+HM** | | | | | **-HM** | | | | | |
| --- | --- | --- | --- | --- | --- | --- | --- | --- | --- | --- | --- | --- |
| ***#COG*** | ***HM genes*** | **1** | **2** | **3** | **4** | **5** | **6** | **7** | **8** | **9** | **10** | **11** |
| COG4986 | ABC-type anion transport system, duplicated permease component | 1 | - | - | - | - | - | - | - | - | - | - |
| COG0310 | ABC-type Co^2+^ transport system, permease component | - | - | - | - | - | - | - | - | 1 | - | - |
| COG1122 | ABC-type cobalt transport system, ATPase component | 1 | 1 | 1 | 1 | 1 | 1 | 1 | 1 | 2 | 2 | 1 |
| COG0619 | ABC-type cobalt transport system, permease component CbiQ and related transporters | 3 | 4 | 3 | 3 | 3 | 4 | 3 | 3 | 4 | 3 | 3 |
| COG0444 | ABC-type dipeptide/oligopeptide/nickel transport system, ATPase component | 10 | 8 | 11 | 9 | 10 | 8 | 9 | 1 | 8 | 6 | 9 |
| COG1124 | ABC-type dipeptide/oligopeptide/nickel transport system, ATPase component | - | 1 | 1 | - | 1 | - | 1 | - | - | - | 1 |
| COG0601 | ABC-type dipeptide/oligopeptide/nickel transport systems, permease components | 17 | 14 | 21 | 16 | 15 | 12 | 13 | 3 | 11 | 11 | 17 |
| COG1173 | ABC-type dipeptide/oligopeptide/nickel transport systems, permease components | 17 | 14 | 21 | 16 | 15 | 12 | 13 | 3 | 11 | 11 | 17 |
| COG1840 | ABC-type Fe^3+^ transport system, periplasmic component | 4 | 3 | 1 | 1 | 2 | 3 | 2 | 2 | 1 | 1 | 2 |
| COG1178 | ABC-type Fe^3+^ transport system, permease component | 3 | 1 | - | - | 1 | 2 | 1 | 1 | - | 1 | 1 |
| COG1135 | ABC-type metal ion transport system, ATPase component | 2 | 1 | 1 | 1 | 1 | 1 | 1 | 1 | 1 | 1 | 1 |
| COG0803 | ABC-type metal ion transport system, periplasmic component/surface adhesin | 1 | 1 | 1 | 1 | 1 | 2 | 2 | 1 | 2 | - | 1 |
| COG1464 | ABC-type metal ion transport system, periplasmic component/surface antigen | 2 | 1 | 1 | 1 | 1 | 1 | 1 | 1 | 1 | 1 | 1 |
| COG2011 | ABC-type metal ion transport system, permease component | 2 | 1 | 1 | 1 | 1 | 1 | 1 | 1 | 1 | 1 | 1 |
| COG1121 | ABC-type Mn/Zn transport systems, ATPase component | 1 | 1 | 1 | 1 | 1 | 2 | 2 | 1 | 2 | 1 | 1 |
| COG1108 | ABC-type Mn^2+^/Zn^2+^ transport systems, permease components | 1 | 1 | 1 | 1 | 1 | 2 | 2 | 1 | 2 | 1 | 1 |
| COG0725 | ABC-type molybdate transport system, periplasmic component | 1 | 2 | 1 | 2 | 1 | 1 | 1 | 1 | 1 | 1 | 1 |
| COG4149 | ABC-type molybdate transport system, permease component | 1 | 1 | 1 | 1 | 1 | - | 1 | 1 | - | - | 1 |
| COG1119 | ABC-type molybdenum transport system, ATPase component/photorepair protein PhrA | 2 | 2 | 1 | 1 | 1 | 2 | 1 | 1 | 1 | 1 | 1 |
| COG4531 | ABC-type Zn^2+^ transport system, periplasmic component/surface adhesin | 1 | - | - | - | - | - | 1 | 2 | - | - | - |
| COG0798 | Arsenite efflux pump ACR3 and related permeases | - | - | 2 | 3 | 1 | - | - | 1 | - | - | - |
| COG0474 | Cation transport ATPase | 1 | - | - | 1 | - | 1 | - | - | 2 | 2 | - |
| COG2217 | Cation transport ATPase | 3 | 2 | 5 | 6 | 2 | 3 | 2 | 6 | 2 | 2 | 2 |
| COG2059 | Chromate transport protein ChrA | - | - | - | 1 | - | - | - | - | - | - | - |
| COG1230 | Co/Zn/Cd efflux system component | 1 | 1 | 3 | 2 | 1 | 1 | 1 | 1 | 1 | 1 | 1 |
| COG2608 | Copper chaperone | 1 | 1 | 3 | 1 | 2 | 1 | 1 | 2 | 1 | 1 | 1 |
| COG0735 | Fe^2+^/Zn^2+^ uptake regulation proteins | 2 | 2 | 2 | 2 | 2 | 2 | 2 | 2 | 2 | 2 | 2 |
| COG0672 | High-affinity Fe^2+^/Pb^2+^ permease | 1 | 1 | 1 | 1 | 1 | 1 | 1 | 1 | - | 1 | 1 |
| COG3376 | High-affinity nickel permease | - | - | - | - | - | - | - | 1 | - | - | - |
| COG0861 | Membrane protein TerC, possibly involved in tellurium resistance | 1 | 1 | 2 | 2 | 2 | 2 | 2 | 2 | 2 | 2 | 2 |
| COG2076 | Membrane transporters of cations and cationic drugs | 3 | 1 | 3 | 2 | 3 | 3 | 1 | - | 1 | 1 | 3 |
| COG2239 | Mg/Co/Ni transporter MgtE (contains CBS domain) | 1 | 1 | 1 | 1 | 1 | 1 | 1 | 2 | 1 | 1 | 1 |
| COG0598 | Mg^2+^ and Co^2+^ transporters | 2 | 3 | 2 | 2 | 1 | 2 | 2 | 1 | 1 | 1 | 2 |
| COG1914 | Mn^2+^ and Fe^2+^ transporters of the NRAMP family | 1 | 2 | 3 | 2 | 2 | 2 | 1 | 2 | 1 | 1 | 2 |
| COG1055 | Na^+^/H^+^ antiporter NhaD and related arsenite permeases | 1 | 1 | 1 | 1 | - | 1 | 1 | 1 | - | - | 2 |
| COG0053 | Predicted Co/Zn/Cd cation transporters | 4 | 4 | 4 | 5 | 4 | 4 | 4 | 4 | 2 | 3 | 4 |
| COG0428 | Predicted divalent heavy-metal cations transporter | - | - | 1 | 1 | 1 | - | 1 | - | 1 | 1 | 1 |
| COG4300 | Predicted permease, cadmium resistance protein | - | - | 1 | 1 | - | - | - | - | - | - | - |
| COG1276 | Putative copper export protein | 1 | 1 | 2 | 2 | 1 | 1 | 1 | 3 | 1 | 1 | 1 |
| COG1275 | Tellurite resistance protein and related permeases | - | - | - | 1 | - | - | - | 1 | - | - | - |
| COG3142 | Uncharacterized protein involved in copper resistance | 2 | 1 | 1 | 1 | 1 | 2 | 1 | - | 1 | 2 | 1 |
|  | **Total** | 95 | 79 | 105 | 94 | 82 | 81 | 78 | 55 | 68 | 64 | 86 |
|  | ***Regulators*** |  |  |  |  |  |  |  |  |  |  |  |
|  | ArsR | 12 | 14 | 9 | 11 | 11 | 8 | 8 | 11 | 7 | 9 | 8 |
|  | MerR | 5 | 6 | 3 | 3 | 8 | 5 | 4 | 4 | 5 | 4 | 6 |
|  | CsoR | 1 |  | 2 | 1 |  | 1 |  | 2 | 1 | 1 | 1 |
|  | Fur | 1 | 1 | 1 | 1 | 1 | 1 | 1 | 1 | 1 | 1 | 1 |
|  | Zur | 1 | 1 | 1 | 1 | 1 | 1 | 1 | 1 | 1 | 1 | 1 |
|  | DtxR/IdeR | 1 | 1 | 1 | 1 | 1 | 1 | 1 | 1 | 1 | 1 | 1 |
|  | **Total** | 21 | 23 | 17 | 18 | 22 | 17 | 15 | 20 | 16 | 17 | 18 |

**Table S11**. Selected parameters of the experimental soil.

| **Characteristic** | **Value** | **Unit** |  |
| --- | --- | --- | --- |
| Texture (sand/silt/clay) | 350/550/100 | g kg^-1^ | |
| Cation exchange capacity (at soil pH) | 247 | mmol kg^-1^ | |
| Organic carbon | 24.6 | g kg^-1^ | |
| pH | 7.2 |  | |
| *Total metal contents (in aqua regia)* | |  | |
| Zn | 1,760 | mg kg^-1^ | |
| Cd | 32.7 | mg kg^-1^ | |
| Pb | 6,560 | mg kg^-1^ | |
| *Mobile fraction of metals (in 1M NH_4_NO_3_)* | | | |
| Zn | 2.56 | mg kg^-1^ | |
| Cd | 0.64 | mg kg^-1^ | |
| Pb | 3.81 | mg kg^-1^ | |

**Figure S1.** Maximum likelihood phylogenetic tree based on 16S rRNA gene sequences. Genome sequences were downloaded from NCBI (May 8^th^, 2020) with ncbi-genome-download tool (Blin, 2020) and data were first filtered according to the number of contigs (<100), genome completeness (>90%) and contamination (<3%) with CheckM (Parks et al., 2014). Sequences of 16S rRNA genes were extracted using barrnap ((Seemann, 2020) and then processed with Metaxa2 (Bengtsson-Palme et al., 2015) to confirm the targeted extraction and check their orientation (Table S2). All copies were used for the construction of the tree. In addition, we included the 16S rRNA gene sequences of the isolates of this study that have no genome available (Table 1). DNA was released by boiling one bacterial colony for 8 min in 50 µl sterile deionized water followed by incubation on ice for 5 min. After centrifugation at 13000 rpm for 10 min, 2 µl of the lysis product were used as template in 20 µl PCR reaction containing one unit FIREpol DNA polymerase (Soils Biodyne), 0.2 mM dNTPs mix, 2.25 mM MgCl_2_ and 0.3 µM of each primer (8f: 5’-AGAGTTTGATCCTGGCTCAG-3’, Weisburg et al.,1991; 1520r: 5’-AAGGAGGTGATCCAGCCGCA-3’, Edwards et al., 1989). The thermal program included an initial denaturation of 5 min at 95°C, 35 cycles of 1 min denaturation at 95°C, 90 sec annealing at 53°C and 2 min elongation at 72°C followed by a final elongation of 10 min at 72°C. After sequencing, the 16S rRNA gene sequences were subjected to BLAST analysis against the GenBank database. The corresponding accession numbers can be found in Table 1 in the main text.

To construct the phylogenetic tree, short fragments were filtered out with an ad hoc Python script. Sequences were first aligned with MAFFT (Katoh et al., 2019), then trimmed with TRIMal (Capella-Gutierrez et al., 2009) and a refined alignment was performed using MUSCLE (Edgar, 2004). Finally, ModelTest-NG (Darriba et al., 2019) was used to identify the best model for the construction of the Maximum-Likelihood tree in RAxML (Kozlov et al., 2019) using the Transfer Bootstrap method for branch support (Lemoine et al., 2018). Bootstrap values > 50% are shown at branch points.

The 70 genomes selected for further analysis are in bold. In the inner ring, strains belonging to functional groups I, II and III are highlighted in green, blue and red, respectively. Strains isolated from heavy metal contaminated sites are marked with a black circle on the corresponding branch. In the second ring, results for the following tests are shown: auxine (IAA), ACC deaminase and siderophore production, phosphate solubilization and resistance to heavy metal (Zn, Pb, Cd).

Bengtsson-Palme J., Hartmann, M., Eriksson, K.M., Pal, C., Thorell, K., Larsson, D.G.J., and Nilsson, R.H. (2015) Metaxa2: Improved Identification and Taxonomic Classification of Small and Large Subunit rRNA in Metagenomic Data. Molecular Ecology Resources: doi: 10.1111/1755-0998.12399

Blin, K. (2020) Ncbi-genome-download, GitHub repository, https://github.com/kblin/ncbi-genome-download.

Capella-Gutierrez; S., Silla-Martinez, J.M., and Gabaldon, T. (2009) trimAl: a tool for automated alignment trimming in large-scale phylogenetic analyses. Bioinformatics, 25: 1972-1973.

Edgar, R.C. (2004) MUSCLE: multiple sequence alignment with high accuracy and high throughput. Nucl Acids Res 32: 1792-1797. doi: 10.1093/nar/gkh340

Edwards, U., Rogall, T., Blocker, H., Emde, M., and Bottger, E.C. (1989) Isolation and direct complete nucleotide determination of entire genes. Characterization of a gene coding for 16S ribosomal RNA. Nucl Acids Res 17: 7843-7853.

Darriba, D., Posada, D., Kozlov, A.M., Stamatakis, A., Morel, B., and Flouri, T. (2019) ModelTest-NG: a new and scalable tool for the selection of DNA and protein evolutionary models. Molecular Biology and Evolution 18. doi:10.1093/molbev/msz189.

Katoh, K., Rozewicki, J., and Yamada, K.D. (2019 ) MAFFT online service: multiple sequence alignment, interactive sequence choice and visualization. Briefings in Bioinformatics, 20: 1160-1166.

Kozlov, A.M., Darriba, D., Flouri, T., Morel, B., and Stamatakis, A. (2019) RAxML-NG: a fast, scalable and user-friendly tool for maximum likelihood phylogenetic inference. Bioinformatics 35: 4453–55. doi:10.1093/bioinformatics/btz305.

Lemoine, F., Domelevo Entfellner, J.-B., Wilkinson, E., Correia, D., Dávila Felipe, M., De Oliveira, T., and Gascuel, O. (2018) Renewing Felsenstein’s phylogenetic bootstrap in the era of big data. Nature 556: 452–456. doi:10.1038/s41586-018-0043-0.

Parks, D.H., Imelfort, M., Skennerton, C.T., Hugenholtz, P., and Tyson, G.W. (2014) Assessing the quality of microbial genomes recovered from isolates, single cells, and metagenomes. Genome Research, 25: 1043-1055.

Seemann, T. (2020) barrnap, GitHub repository, https://github.com/tseemann/barrnap.

Weisburg, W.G., Barns, S.M., Pelletier, D.A., and Lane, D.J. (1991) 16S ribosomal DNA amplification for phylogenetic study. J Bacteriol 173: 697-703.

**Figure S2**. *Microbacterium* pan-genome calculated in Roary (Page et al., 2015). This graph shows how the pan-genome varies as new genomes are added in random order.

**Figure S3.** Maximum likelihood phylogenetic trees based on the main biosynthetic genes of the terpenoid gene cluster: lycopene elongase **(A)**, geranylgeranyl-pyrophosphate (GGPP) synthase **(B)**, C_50_ carotenoid cyclases crtY_e/f_ **(C)** and crtY_g/h_ **(D)**. Bootstrap values > 50% are shown at branch points. Strains belonging to the terpenoid cluster group A are marked in blue; whereas the ones belonging to group B are marked in red. Isolates in bold were tested in this study. Isolates marked with a black circle were isolated from heavy metal contaminated sites.

**Figure S4.** Maximum likelihood phylogenetic tree based on glycosyl transferases related to the terpenoid gene cluster. Bootstrap values > 50% are shown at branch points. Strains belonging to the phylogenetic group A are marked in blue; whereas the ones belonging to group B are marked in red. Isolates in bold were tested in this study. Isolates marked with a black circle were isolated from heavy metal contaminated sites.

For some *Microbacterium* genomes (*M. ketosireducens* DSM 12510, *M. azadirachtae* DSM23848, *M. oxydans* BEL4b and *M. hydrocarbonoxydans* SA35), the selected glycosyl transferases form a separate group (flanked by question mark “?”) because of the low sequence homology with the one of *Micrococcus luteus* NCTC 2665, suggesting that they might be involved in different glycosylation processes.

**Figure S5.** Chromophore of yellow pigment of *Microbaceterium* sp. 1.5R and C_50_ carotenoids found in genera with related carotenoid biosynthesis genes.

Briefly, 200mg bacteria of *Microbaceterium* sp. 1.5R, *M. azadirachtae* ARN176 and *M.* *ginsengisoli* DSM 18659 were scrapped from a plate, re-suspended in 1 ml water, washed with 800 µl chloroform (pale chloroform obtained here was discarded), pelleted (3000 rpm, 5 min) and extracted with 1 ml MeOH. MeOH was evaporated in speedvac, samples were dissolved in 500 µl water. 250 µl of this was acidified with HCl (1% final concentration), hydrolyzed at 70°C for 30 min, neutralized with 2 N NaOH and extracted with 500 µl chloroform. The fraction was dried and dissolved in 200 µl MeOH. 2 µl were used to record UV-VIS spectra showing a nona-en chromophore (max at 415, 440, 470 nm) for *Microbaceterium* sp. 1.5R **(A)** and *M.* *ginsengisoli* DSM18659 (not shown). Extracts of the colorless strain *M. azadirachtae* ARN176 does not show a characteristic UV-VIS spectrum **(A)**. The chemical properties (hydrophilic before, lipophilic after hydrolysis), the color and the UV-VIS spectrum and the carotenoid biosynthesis clusters found in the genome point to structures similar or identical to glycosylated sarcinaxanthin or decaprenoxanthin characterized in *Micrococcus luteus* (Netzer et al., 2010) and *Corynebacterium glutamicum* (Heider et al., 2012), respectively **(B)**.

**Figure S6.** Maximum likelihood phylogenetic tree based on non-ribosomal peptide synthetase (NRPS) gene sequences. Bootstrap values > 50% are shown at branch points. Strains belonging to phylogenetic groups I, II and III are highlighted in green, blue and red, respectively. Isolates in bold were tested in this study. Isolates marked with a black circle were isolated from heavy metal contaminated sites. Type A NRPSs (yellow) bind glycine, alanine or a hydrophilic compound as substrate; type B1/2 NRPSs (purple/lilac) bind negatively charged (asp, glu) and uncharged polar (asn, gln) amino acids and 2-amino-adipic acid, respectively; type C NRPSs (brown) bind hydrophobic or aliphatic amino acids.

**Figure S7.** Maximum likelihood phylogenetic tree based on type III polyketide synthase (PKS III). Bootstrap values > 50% are shown at branch points. Isolates in bold were tested in this study. Isolates marked with a black circle were isolated from heavy metal contaminated sites.

*Microbacterium* sp. Root61 clusters within the PKS type A1 cluster, but contains a type A2 long-chain fatty acid-CoA ligase (FadD15). The other members of PKS cluster type A2 are *Microbacterium* sp. UNCCL10 and *M. chocolatum* SIT101, belonging to the phylogenetic group II, and *M. mangrovi* MUSC115, which is the only strain of the phylogenetic group III harboring a PKS cluster.

**Figure S8.** Maximum likelihood phylogenetic tree based on long-chain fatty acid-CoA ligase (FadD15) gene sequences of the polyketide synthase (PKS) clusters. Bootstrap values > 50% are shown at branch points. Isolates in bold were tested in this study. Isolates marked with a black circle were isolated from heavy metal contaminated sites.

**Figure S9.** Mobilization of copper **(A)** and manganese **(B)** from contaminated soil by bacterial exudates. The different colors of the diagram bars represent different isolation sources: shoots in light green; roots in dark green; rhizosphere in orange; soil in brown; nodules in dark red. NC, negative control (gray) is non-inoculated medium. +HM: bacteria isolated from heavy metal contaminated sites; -HM: bacteria isolated from non-contaminated sited. Error bars show standard errors of the mean (n = 6, except for NC n = 3 and for bacteria producing a polysaccharide matrix difficult to filtrate: n = 5 for EX104 and 280; n = 3 for 228 and K02; n = 2 for K01; n = 1 for DSM 8608). Samples with extraction values differing significantly from the control (NC) are labelled with * (p < 0.05) and ** (p < 0.01). The order of magnitude of CFU/mL prior to filtration are indicated above the diagram bars.

**Figure S10.** The effect of pH on the mobilization of zinc **(A)**, cadmium **(B)**, lead **(C)**, iron **(D)**, copper **(E)** and manganese **(F)** from contaminated soil by bacterial exudates. Black dots represent non-inoculated medium adjusted at different pH values; red dots represent the total amount of mobilized metal by the different bacterial filtrates and their corresponding pH value prior incubation with the contaminated soil.

**Figure S11.** Dry biomass of *B. napus* roots and shoots **(A)** and *N. caerulescens* **(B)** shoots. Briefly, after sterilization (20 min in 50% sodium hypochlorite with 0.05% Tween 20, 1 min in 70% EtOH, wash 6 times in sterile deionized water), *N. caerulescens* seeds were placed on Murashige and Skoog (MS) agar medium (4.31 g l^-1^ MS, 0.5 g l^-1^ MES 2-(N-morpholino)ethanesulfonic acid, 10 g l^-1^ agar, pH 5.7) and incubated for 5 days at 4°C, then transferred at room temperature in the light for germination. *B. napus* seeds were placed directly on wetted filter paper at room temperature in the light for germination. Seedlings were transferred in pots containing the same contaminated soil as used in the extraction assay (Table S8) (Kuffner et al., 2010). Plants were inoculated once a week for 3 weeks with bacterial suspension. Bacteria were grown in Landy medium at 27°C and 200 rpm till the late logarithmic phase, then harvested by centrifugation (20 min, 4700 rpm, 4°C) and resuspended in potassium phosphate buffer 50 mM pH 7 to a final concentration of about 10^8-9^ CFU/ml. Four weeks after the first inoculation, shoots were harvested, washed with deionized water and dried at 70°C for 48h. A subsample (0.2 g) of the dried and ground plant material was digested in a mixture of concentrated HNO_3_ (65%, 5 ml) and H_2_O_2_ (30%, 1 ml) using an automated heating block. Digests were diluted to reach a matrix of 2% HNO_3_ and analyzed for metal concentrations by ICP-MS (Elan DRCe 9000, Perkin Elmer). NC, negative control: non-inoculated plants; treatments ARN176, 1.5R and SA35: plants were inoculated with *M. azadirachtae* ARN176, *Microbacterium* sp. 1.5R and *M. hydrocarbonoxydans* SA35, respectively.

**Figure S12.** Concentration of zinc, cadmium, lead, iron, manganese **(A, B)** and copper **(C)** in *N. caerulescens* and *B. napus* shoots after inoculation with *M. azadirachtae* ARN176 (poor mobilizer), *Microbacterium* sp. 1.5R and *M. hydrocarbonoxydans* SA35 (two best mobilizers) in the experiment shown in Figure S11.

NC, non-inoculated plants. Error bars show standard error of the mean (n = 6 for *N. caerulescens*; n = 10 for *B. napus*). Values differing significantly from the control (NC) are labelled with * (p < 0.05) and ** (p < 0.01).

**Figure S13.** Concentration of zinc, cadmium, lead, iron, manganese and copper in *B. napus* roots after inoculation with *M. azadirachtae* ARN176 (poor mobilizer), *Microbacterium* sp. 1.5R and *M. hydrocarbonoxydans* SA35 (two best mobilizers) in the experiment shown in Figure S11. NC, non-inoculated plants. Error bars show standard error of the mean (n = 10 for *B. napus*). Values differing significantly from the control (NC) are labelled with * (p < 0.05) and ** (p < 0.01).
