# Supplementary material for: Comparative Genomics of Microbacterium Species to Reveal Diversity, Potential for Secondary Metabolites and Heavy Metal Resistance
Source: Front Microbiol. 2020 Aug 4;11:1869. doi: 10.3389/fmicb.2020.01869 (PMC7438953; doi:10.3389/fmicb.2020.01869)
Supplement: Supplementary file 2 [file Presentation_1.PPTX]

## Slide 1
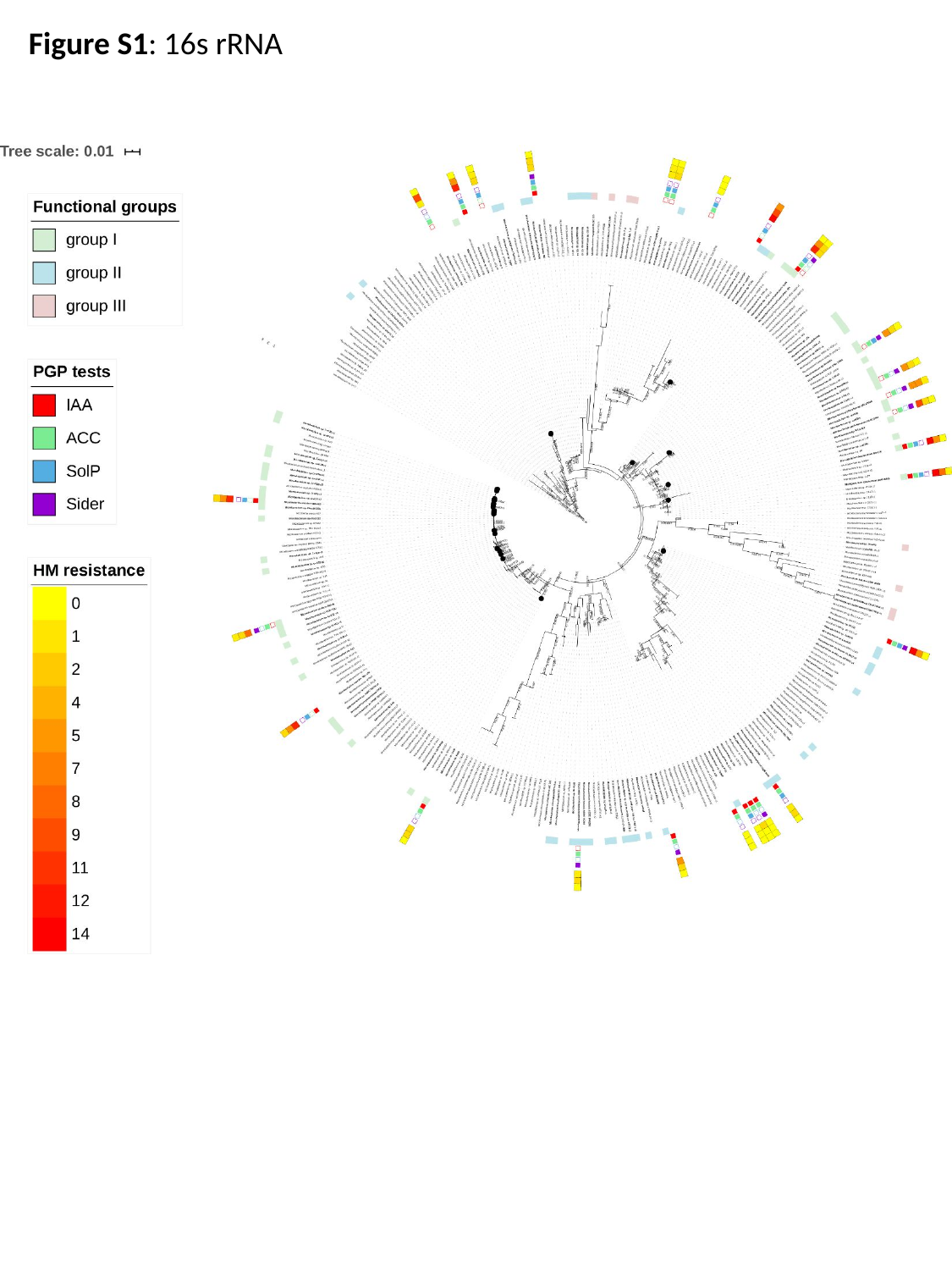

Figure S1: 16s rRNA

## Slide 2
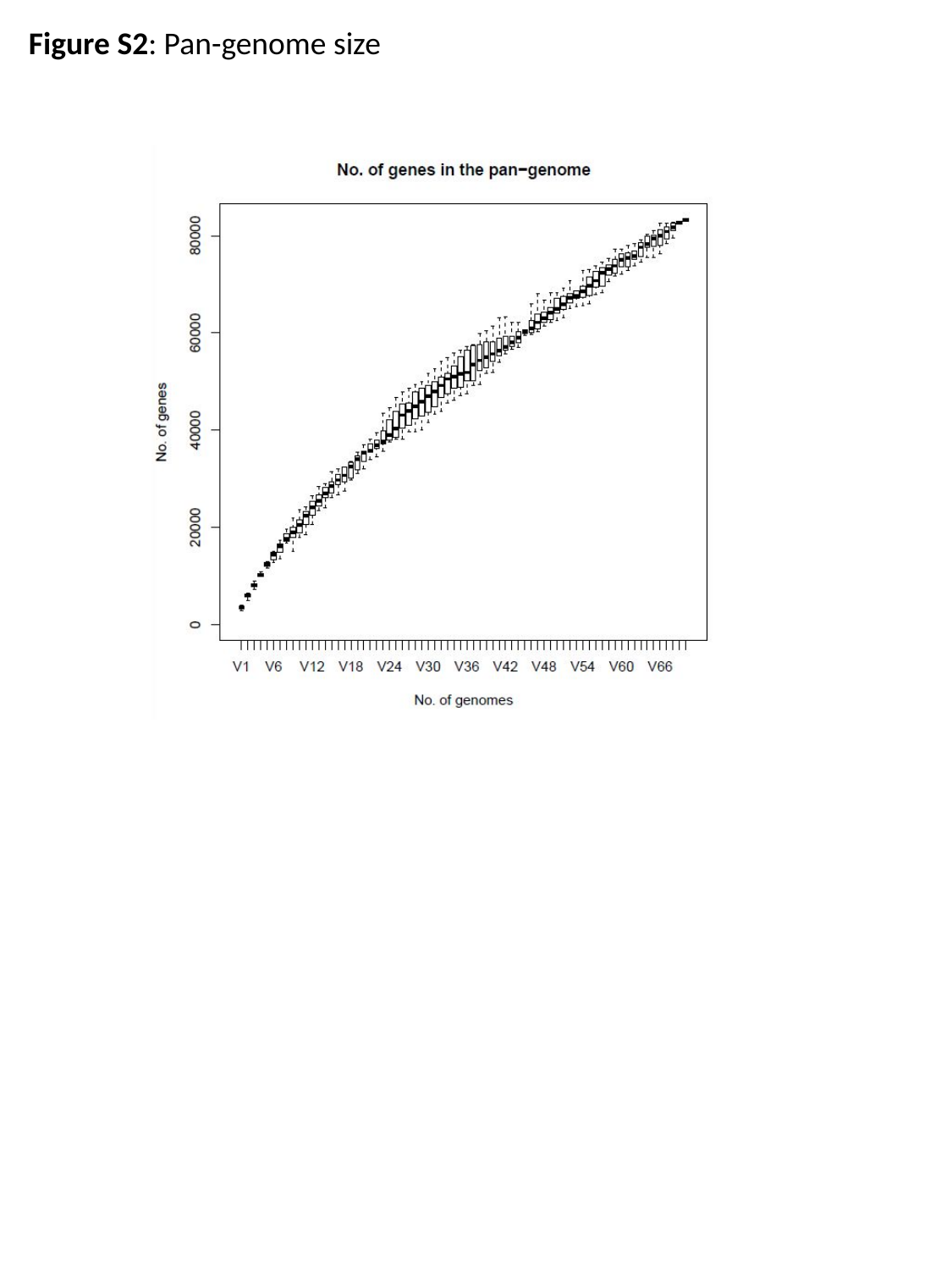

Figure S2: Pan-genome size

## Slide 3
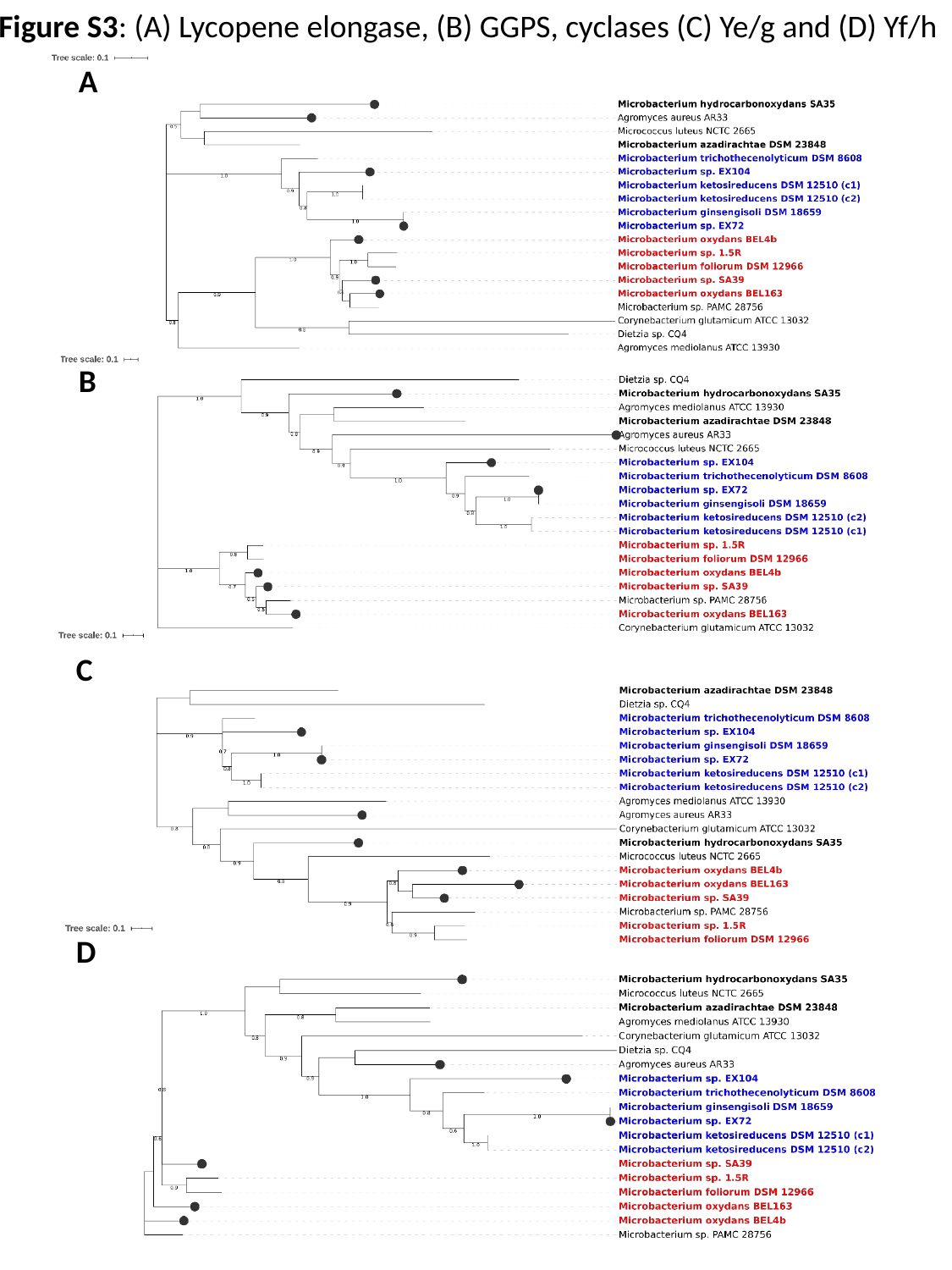

Figure S3: (A) Lycopene elongase, (B) GGPS, cyclases (C) Ye/g and (D) Yf/h
A
B
C
D

## Slide 4
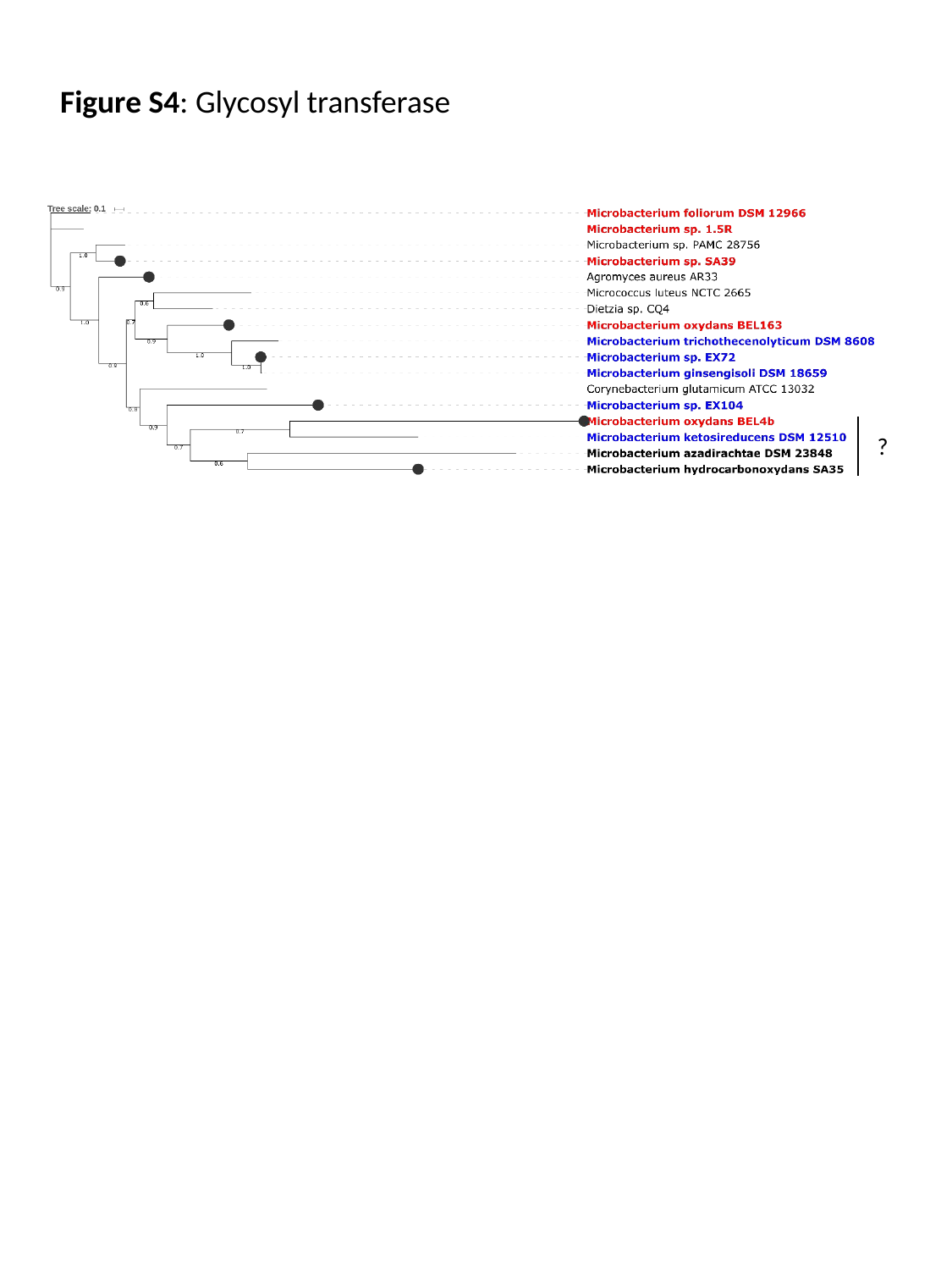

Figure S4: Glycosyl transferase
?

## Slide 5
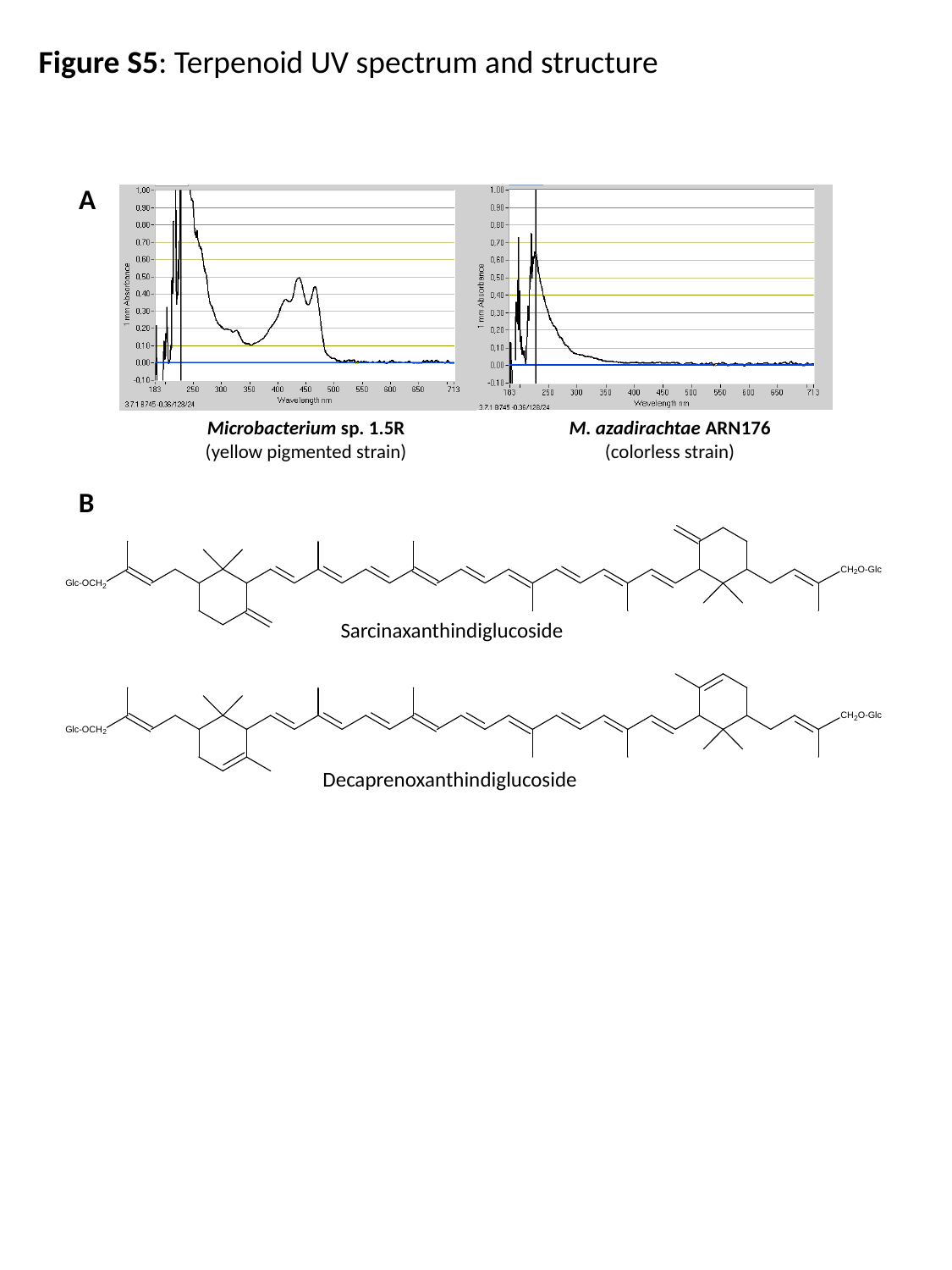

Figure S5: Terpenoid UV spectrum and structure
A
Microbacterium sp. 1.5R
(yellow pigmented strain)
M. azadirachtae ARN176
(colorless strain)
B
Sarcinaxanthindiglucoside
Decaprenoxanthindiglucoside

## Slide 6
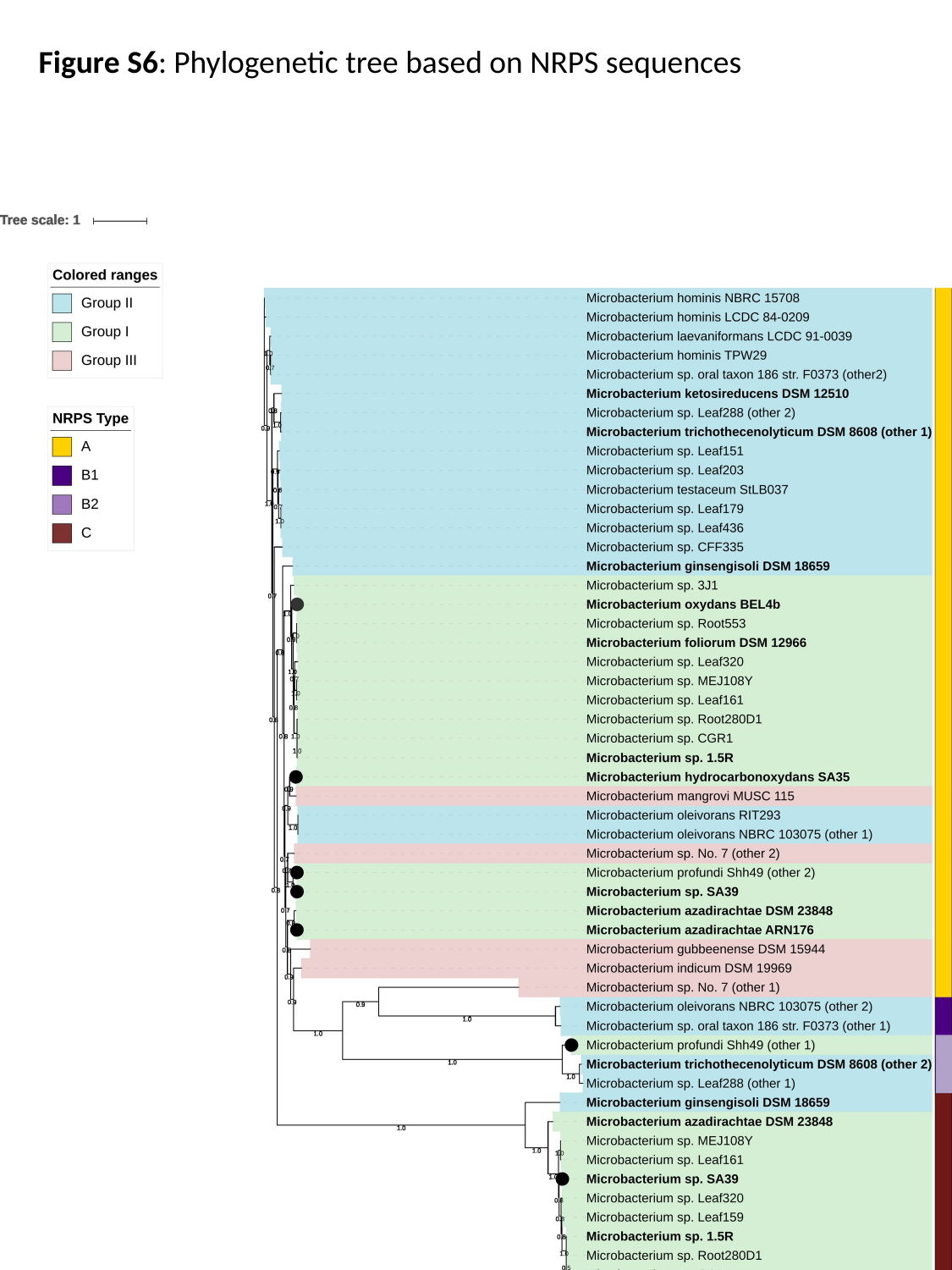

Figure S6: Phylogenetic tree based on NRPS sequences

## Slide 7
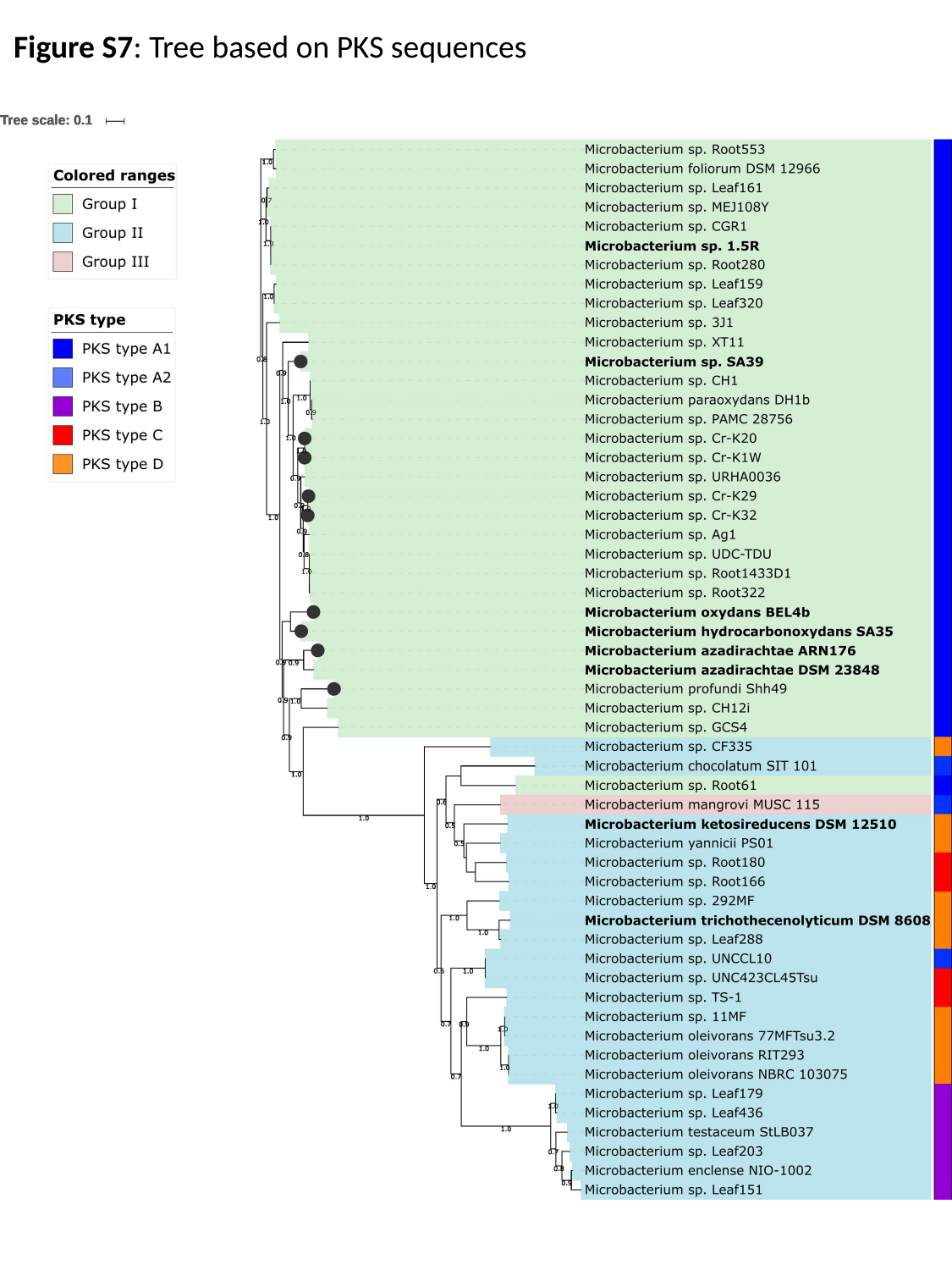

Figure S7: Tree based on PKS sequences

## Slide 8
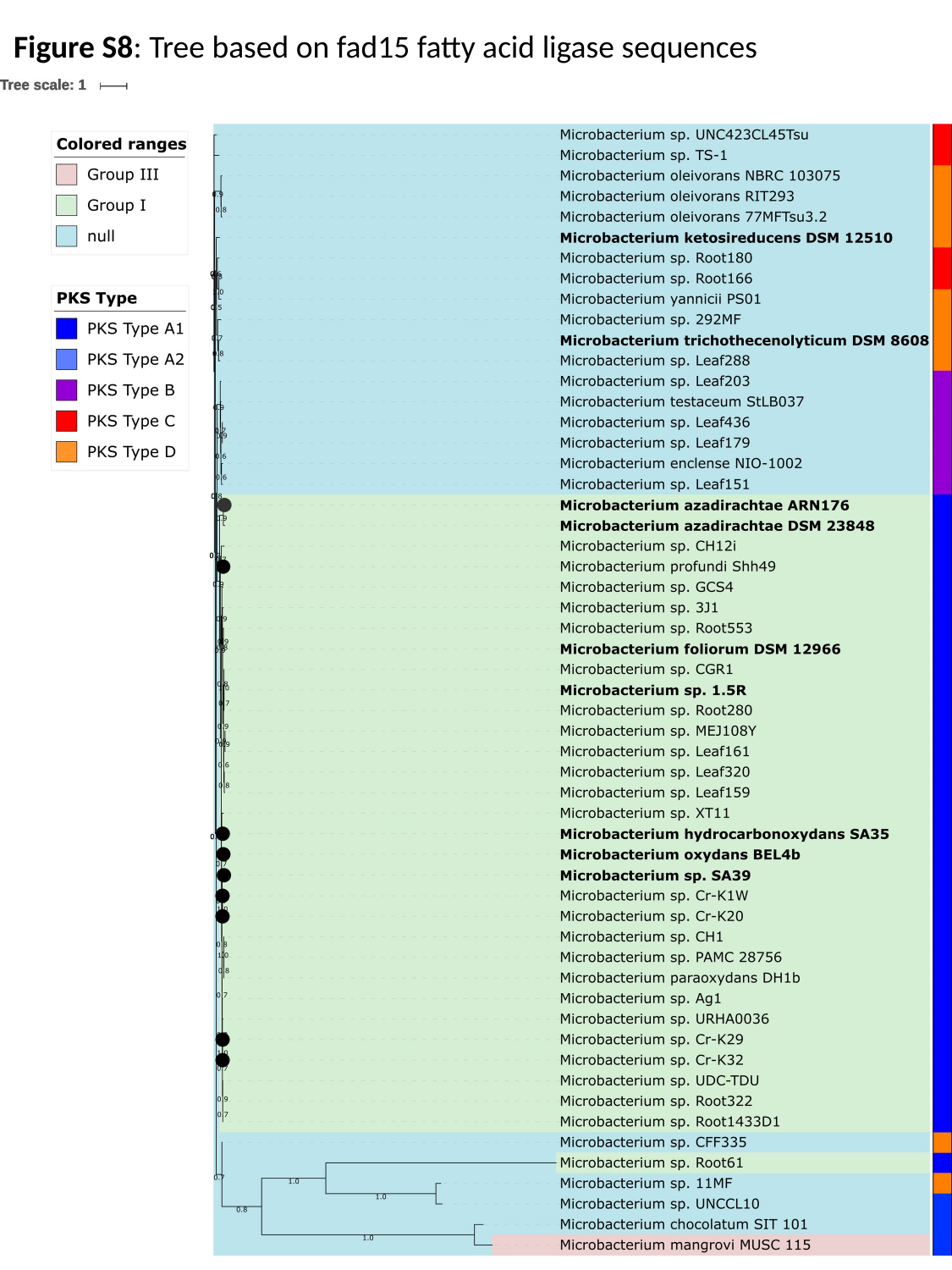

Figure S8: Tree based on fad15 fatty acid ligase sequences

## Slide 9
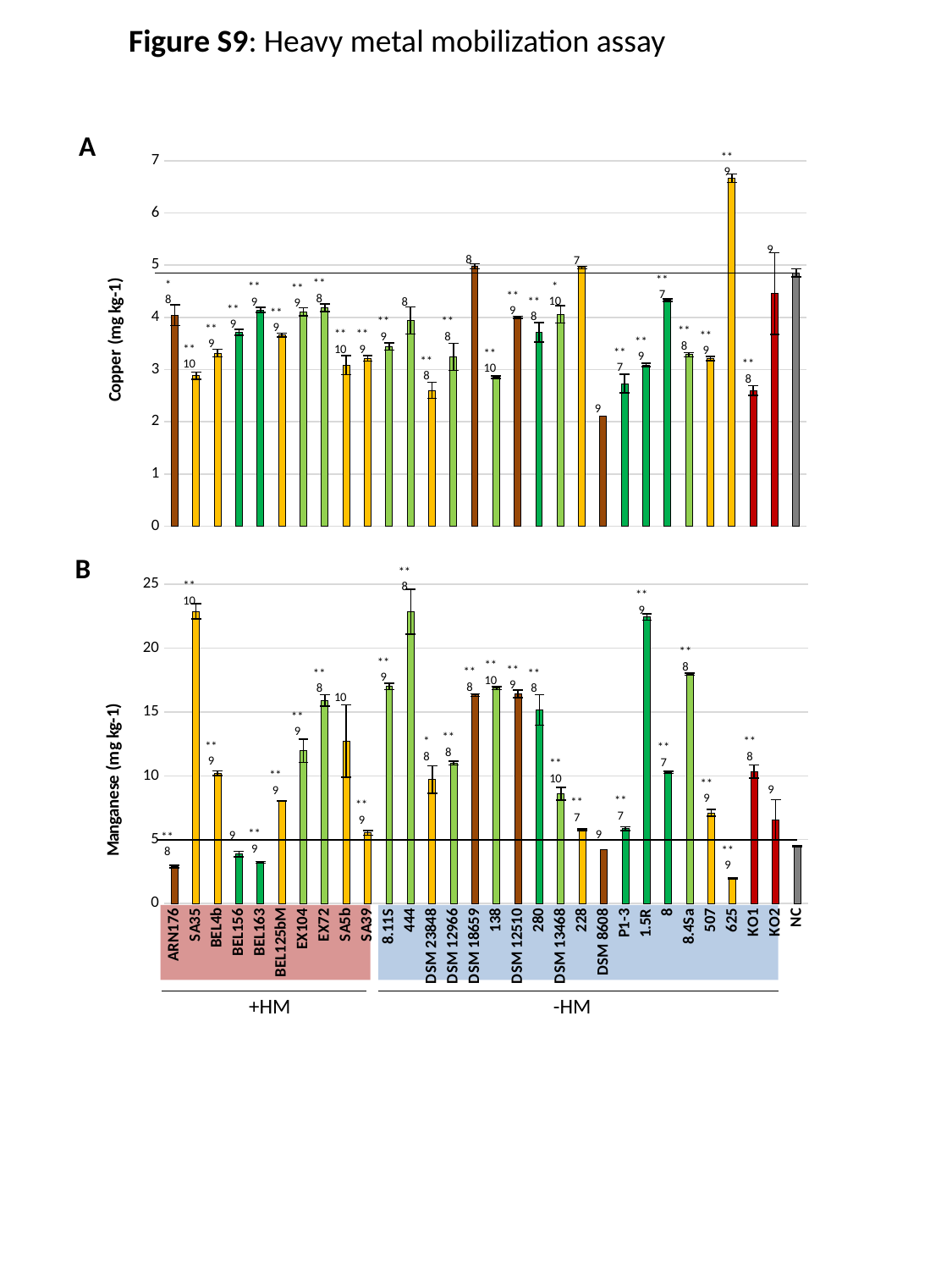

Figure S9: Heavy metal mobilization assay
A
### Chart
| Category | Cu |
|---|---|
| ARN176 | 4.045054270485272 |
| SA35 | 2.8830514439034274 |
| BEL4b | 3.315343534377998 |
| BEL156 | 3.711759299575995 |
| BEL163 | 4.143357011019979 |
| BEL125bM | 3.6576859410680806 |
| EX104 | 4.106544490126743 |
| EX72 | 4.188759456806874 |
| SA5b | 3.0858137649857795 |
| SA39 | 3.2120390874691584 |
| 8.11S | 3.440382920932116 |
| 444 | 3.9420199147301838 |
| DSM 23848 | 2.5995400002155225 |
| DSM 12966 | 3.2434522944179043 |
| DSM 18659 | 4.98024500630694 |
| 138 | 2.853775777466086 |
| DSM 12510 | 4.001293720220448 |
| 280 | 3.715216310746534 |
| DSM 13468 | 4.0571201111119874 |
| 228 | 4.956374451403119 |
| DSM 8608 | 2.112340518627451 |
| P1-3 | 2.732200340803883 |
| 1.5R | 3.0866460620014524 |
| 8 | 4.333599042103237 |
| 8.4Sa | 3.2831666673189823 |
| 507 | 3.2069770308143917 |
| 625 | 6.6648197744976185 |
| KO1 | 2.5960428596663396 |
| KO2 | 4.4587531295729255 |
| NC | 4.854088426356114 |B
### Chart
| Category | Mn |
|---|---|
| ARN176 | 2.8811652245257924 |
| SA35 | 22.85925914457636 |
| BEL4b | 10.214372624577594 |
| BEL156 | 3.869583444553163 |
| BEL163 | 3.217901844486334 |
| BEL125bM | 8.021592763576324 |
| EX104 | 11.955323540647468 |
| EX72 | 15.904226627236048 |
| SA5b | 12.726378698810478 |
| SA39 | 5.514126254623641 |
| 8.11S | 16.97802215183268 |
| 444 | 22.849025826317103 |
| DSM 23848 | 9.692626190051902 |
| DSM 12966 | 10.997664463081486 |
| DSM 18659 | 16.31518960641497 |
| 138 | 16.86227072226025 |
| DSM 12510 | 16.419702751088643 |
| 280 | 15.154413987924624 |
| DSM 13468 | 8.580809702190539 |
| 228 | 5.768601878511313 |
| DSM 8608 | 4.188699228431373 |
| P1-3 | 5.861264128858831 |
| 1.5R | 22.41327028689469 |
| 8 | 10.27049428133874 |
| 8.4Sa | 17.963859462818007 |
| 507 | 7.094117804638869 |
| 625 | 1.9494220890040397 |
| KO1 | 10.330931237487736 |
| KO2 | 6.550051006954837 |
| NC | 4.493902416139697 |+HM
-HM
**
8
*
8
**
10
**
10
**
9
**
9
**
9
9
**
9
**
9
**
9
**
9
**
9
**
9
**
8
**
8
**
10
10
**
9
**
9
**
9
**
9
8
**
8
**8
*
8
**8
**
8
8
**
8
**10
**
10
**
9
**
9
**
8
**
8
*
10
**
10
7
**
7
9
9
**
7
**
7
**
9
**
9
**
7
**
7
**
8
**
8
**
9
**
9
**
9
**
9
**
8
**
8
9
9

## Slide 10
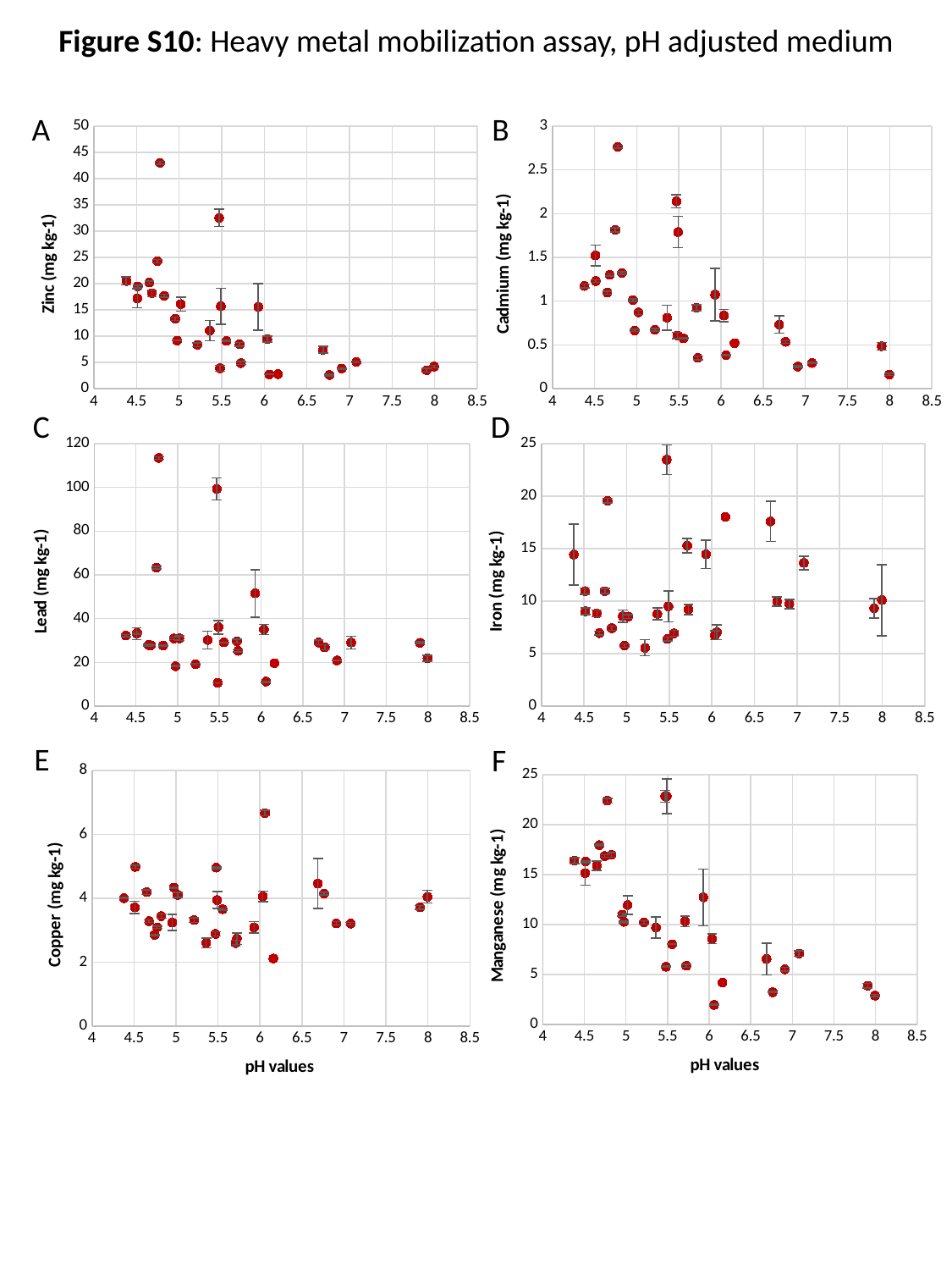

Figure S10: Heavy metal mobilization assay, pH adjusted medium
B
A
### Chart
| Category | | |
|---|---|---|
### Chart
| Category | | |
|---|---|---|
### Chart
| Category | | |
|---|---|---|
### Chart
| Category | | |
|---|---|---|
### Chart
| Category | | |
|---|---|---|
### Chart
| Category | | |
|---|---|---|C
D
E
F

## Slide 11
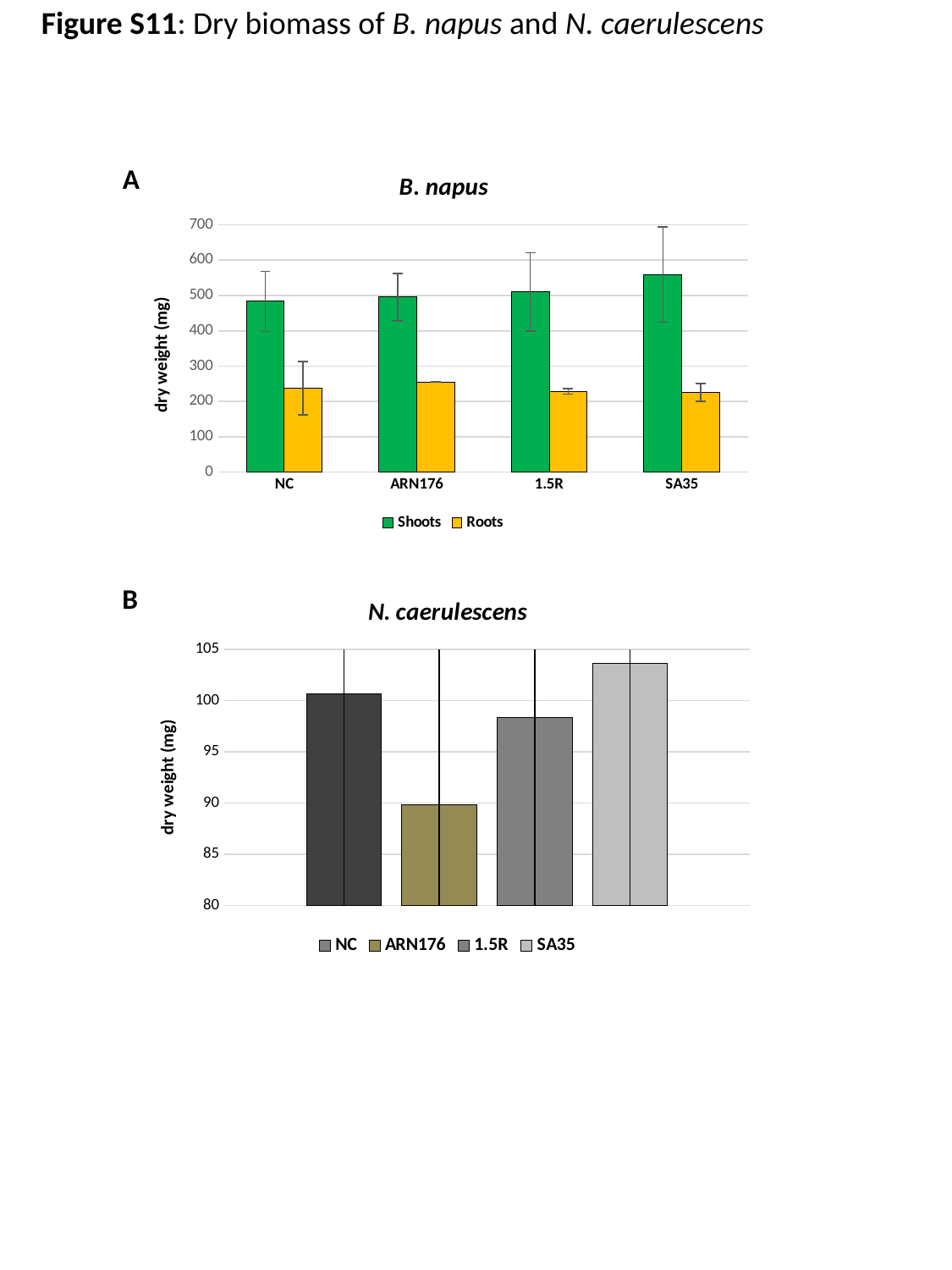

Figure S11: Dry biomass of B. napus and N. caerulescens
A
B
### Chart: N. caerulescens
| Category | NC | ARN176 | 1.5R | SA35 |
|---|---|---|---|---|
### Chart: B. napus
| Category | Shoots | Roots |
|---|---|---|
| NC | 483.3 | 237.5 |
| ARN176 | 495.5 | 255.5 |
| 1.5R | 510.4 | 229.0 |
| SA35 | 559.2 | 225.5 |

## Slide 12
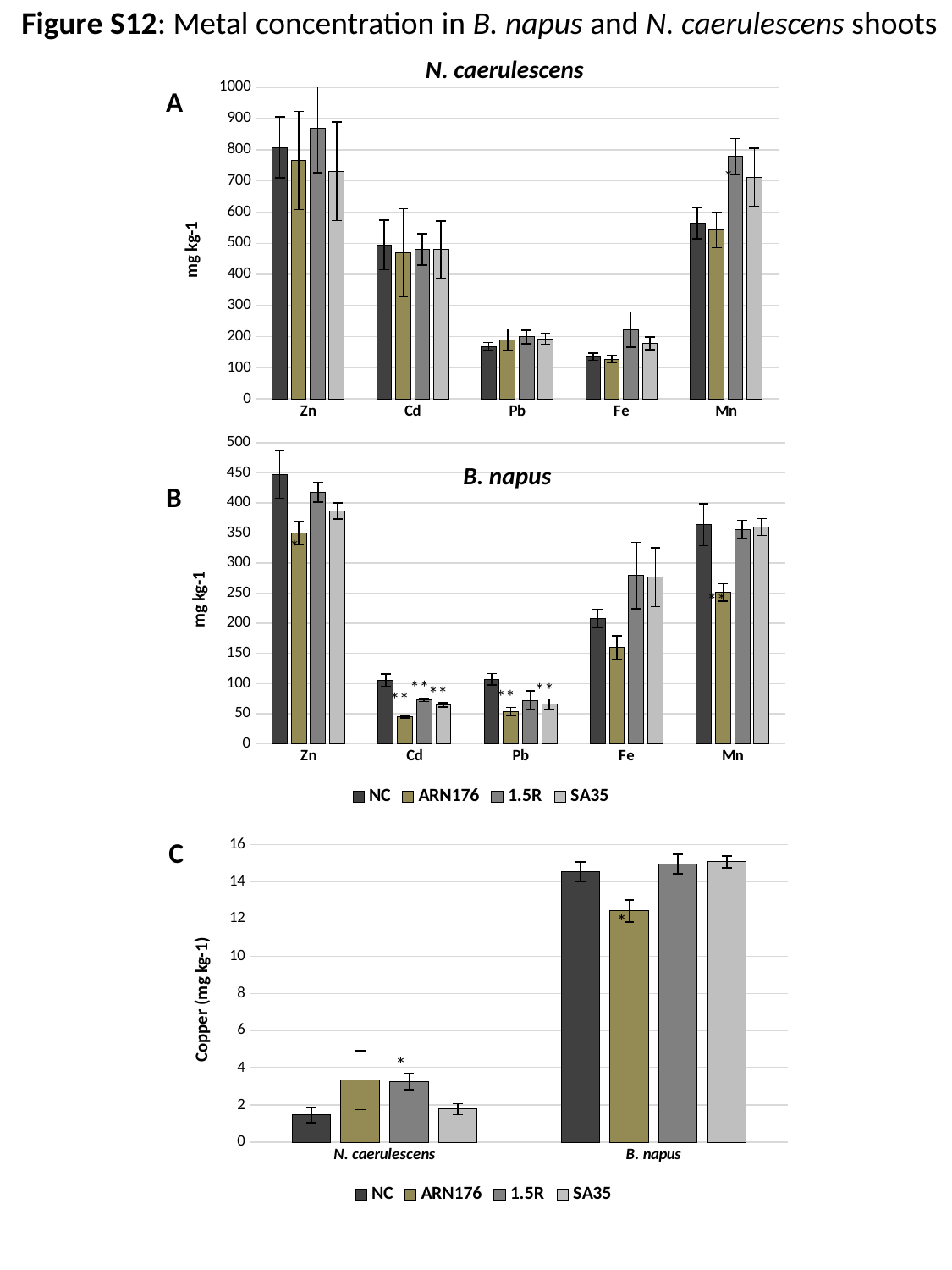

Figure S12: Metal concentration in B. napus and N. caerulescens shoots
N. caerulescens
### Chart
| Category | NC | ARN176 | 1.5R | SA35 |
|---|---|---|---|---|
| Zn | 807.7552514137855 | 765.8649315849055 | 867.900383364211 | 731.1433553386192 |
| Cd | 494.8962053097072 | 469.6383908378727 | 479.9702510952748 | 479.42546145671184 |
| Pb | 168.99661147928978 | 190.3275451682099 | 199.10829553025397 | 192.57663271724695 |
| Fe | 135.74368078155865 | 128.17998369440662 | 222.61846910655117 | 178.72200452671518 |
| Mn | 564.9183833777062 | 541.8931536423135 | 778.3328257993985 | 712.0139098998056 |
### Chart
| Category | NC | ARN176 | 1.5R | SA35 |
|---|---|---|---|---|
| Zn | 447.20248366682335 | 350.0082034762666 | 418.02575774105253 | 386.7389661994977 |
| Cd | 105.2805866395461 | 44.83435389568809 | 72.86246002517079 | 64.62314417730515 |
| Pb | 107.08339049135586 | 54.02586294058428 | 72.09209913373984 | 65.61528889769727 |
| Fe | 208.36319534721127 | 159.6876298798467 | 279.4874540529261 | 276.5809857609112 |
| Mn | 363.5465026529724 | 251.14219378326067 | 356.1055532606282 | 360.09649214757235 |A
B
*
*
**
**
**
**
**
**
B. napus
C
### Chart
| Category | NC | ARN176 | 1.5R | SA35 |
|---|---|---|---|---|
| N. caerulescens | 1.4670266118480664 | 3.3394692665162977 | 3.2608255426626584 | 1.7813745933339418 |
| B. napus | 14.544426517832374 | 12.439940475505622 | 14.949298566145668 | 15.068882887891363 |*
*

## Slide 13
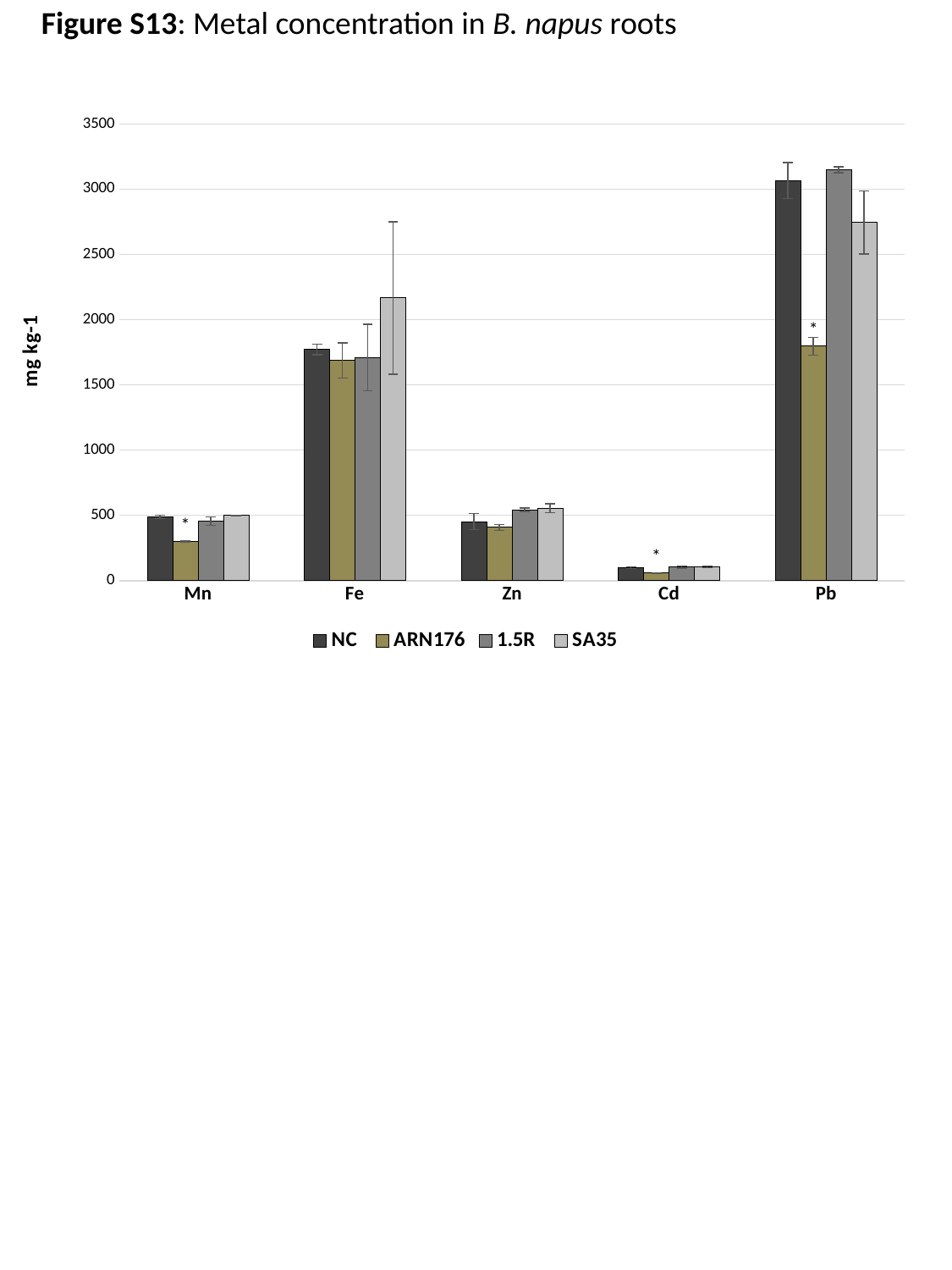

Figure S13: Metal concentration in B. napus roots
### Chart
| Category | NC | ARN176 | 1.5R | SA35 |
|---|---|---|---|---|
| Mn | 487.6229769772359 | 297.8837224998829 | 456.0440956021529 | 499.83663414011426 |
| Fe | 1770.6856302325205 | 1686.648011148718 | 1708.0719987089922 | 2166.0860347968146 |
| Zn | 451.87028786747976 | 407.93939039115355 | 543.1049053318923 | 553.8461618835686 |
| Cd | 99.54576903658537 | 56.80812089541496 | 103.94500789092774 | 105.56155540147178 |
| Pb | 3064.8067183065045 | 1796.546780620979 | 3148.9417475859154 | 2745.2634115764386 |
